# Collection of human reaction times and supporting health related data for analysis of cognitive and physical performance

**DOI:** 10.1016/j.dib.2018.01.025

**Published:** 2018-02-01

**Authors:** Petr Brůha, Roman Mouček, Vítězslav Vacek, Pavel Šnejdar, Kateřina Černá, Petr Řehoř

**Affiliations:** a Department of Computer Science and Engineering, Faculty of Applied Sciences, University of West Bohemia, Univerzitní 8, Pilsen, Czech Republic; b NTIS - New Technologies for the Information Society, Faculty of Applied Sciences, University of West Bohemia, Univerzitní 8, Pilsen, Czech Republic

**Keywords:** Reaction time, Health related data, Cognitive and physical performance, Chronic disease, Data acquisition, Data collection, Software for data collection

## Abstract

Smoking, excessive drinking, overeating and physical inactivity are well-established risk factors decreasing human physical performance. Moreover, epidemiological work has identified modifiable lifestyle factors, such as poor diet and physical and cognitive inactivity that are associated with the risk of reduced cognitive performance. Definition, collection and annotation of human reaction times and suitable health related data and metadata provides researchers with a necessary source for further analysis of human physical and cognitive performance. The collection of human reaction times and supporting health related data was obtained from two groups comprising together 349 people of all ages - the visitors of the Days of Science and Technology 2016 held on the Pilsen central square and members of the Mensa Czech Republic visiting the neuroinformatics lab at the University of West Bohemia. Each provided dataset contains a complete or partial set of data obtained from the following measurements: hands and legs reaction times, color vision, spirometry, electrocardiography, blood pressure, blood glucose, body proportions and flexibility. It also provides a sufficient set of metadata (age, gender and summary of the participant's current life style and health) to allow researchers to perform further analysis. This article has two main aims. The first aim is to provide a well annotated collection of human reaction times and health related data that is suitable for further analysis of lifestyle and human cognitive and physical performance. This data collection is complemented with a preliminarily statistical evaluation. The second aim is to present a procedure of efficient acquisition of human reaction times and supporting health related data in non-lab and lab conditions.

## Specifications table

**Table:** 

**Subject Area**	*Informatics, Biology*
**More specific subject area**	*Health informatics, Human health related data, Infrastructure for health related data collection*
**Type of data**	*Table, graph*
	*Body proportions (Medisana BS 440 Connect),*
	*Electrocardiography (ReadMyHeart Handheld ECG),*
	*Blood pressure (Omron M6 Comfort IT),*
	*Blood sugar (FORA Diamond Mini),*
**How data was acquired**	*Spirometry (Spirometer SP10W)*,
	*Hand reaction time (Device for cognitive research),*
	*Legs reaction time (Impact Dance Pad),*
	*Flexibility (Podium and ruler),*
	*Color vision (Pseudoisochromatic pictures)*
**Data format**	*Raw, preliminary statistically analyzed*
**Experimental factors**	*A custom hardware device was developed for measuring hands reaction times**[1]*. *A custom software tool was developed for measuring legs reaction times**[2]*. *A software infrastructure for rapid collection of health related data was developed**[3]*. *Prior to measurements all participants were familiarized with the goal of the project, overall experimental procedure and related legal conditions.*
**Experimental features**	*Two groups of participants, 349 people of all ages, provided health related data and metadata during the following measurements: hands and legs reaction times, color vision, spirometry, electrocardiography, blood pressure, blood glucose, body proportions, and flexibility. The collected medatata set included age, gender and summary of the participant's current life style and health.*
**Data source location**	*Pilsen, Czech Republic*
	• *Pilsen central square (GPS [49.747285N, 13.377444E]),*
	• *University of West Bohemia (GPS [49.726259N, 13.351782E]).*
**Data accessibility**	*The data are with this article*.
**Related research article**	*The related research article is Exercise and Wellness Health Strategy Framework Software Prototype for Rapid Collection and Storage of Heterogeneous Health Related Data**[3]*.

## Value of data

• The human reaction times and other health related data and metadata were collected from 349 people of all ages.
• There are projects utilizing reaction time as a physiological measure (e.g. [4], [5], [6]) but to the authors best knowledge, there are no datasets publicly available that contain reaction time data together with other supportive health related data and metadata.
• The resulting data collection allows other researchers to perform further analysis, e.g. to detect early symptoms of starting chronic diseases [7], [8].
• The effects of a healthy lifestyle on cognitive functions are of interest to many people.

## 1 Data

The purpose of this article is to provide interested researchers with a well annotated and sufficiently large collection of human reaction times and health related data and metadata that could be suitable for further analysis of lifestyle and human cognitive and physical performance. The second aim is to present a procedure of efficient acquisition of human reaction times and supporting health related data in non-lab and lab conditions.

Each provided dataset contains a complete or partial set of data obtained from the following measurements: hands and legs reaction times, color vision, spirometry, electrocardiography, blood pressure, blood glucose, body proportions and flexibility. It also provides a sufficient set of metadata (age, gender and summary of the participant's current life style and health) to allow researchers to perform further analysis.

## 2 Experimental design, materials and methods

### 2.1 Participants and environment

**Fig. 1:**
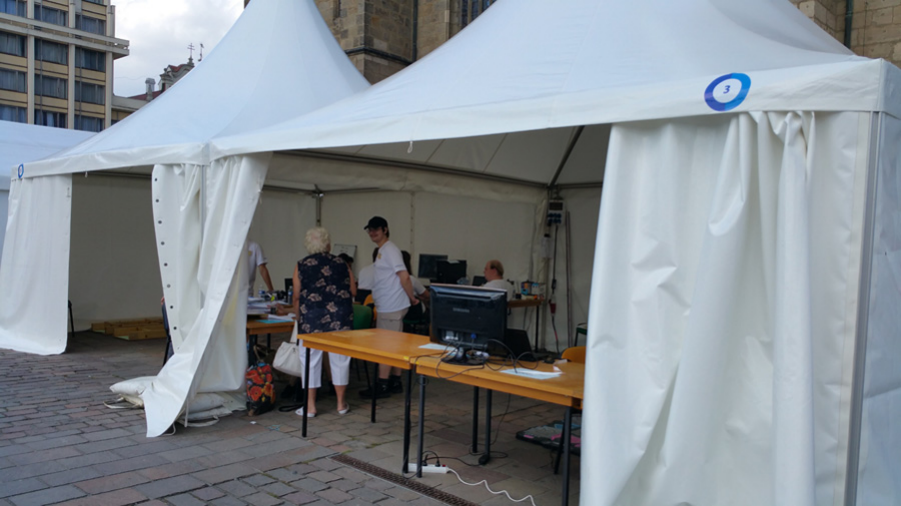
Days of Science and Technology 2016, Pilsen central square, the tent where the measurements were held.

Two groups of participants took part in the project. The first group included 293 people (98 males, 136 females, 59 with no record of their gender in the registration form) visiting the regional event ‘Days of Science and Technology 2016’ held on the central square in Pilsen, Czech Republic in September 2016. The participants were measured in a large textile tent (see Fig. 1). The weather was sunny with an average outside temperature of about 30°C.

**Fig. 2:**
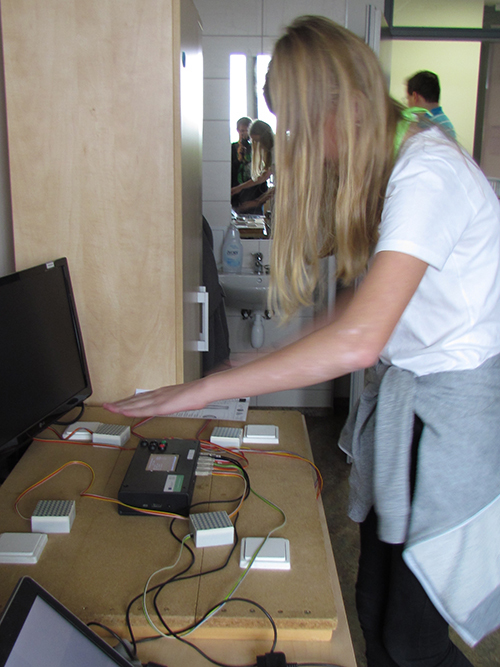
Neuroinformatics laboratory, University of West Bohemia, a participant during measurement of hands reaction time.

The second group of participants included 56 people from the organization Mensa Czech Republic that brings together people with an IQ greater than 130 (30 males, 23 females, 3 with no record of their gender in the registration form). In this case the experiments were performed in the air-conditioned neuroinformatics laboratory, University of West Bohemia, Czech Republic (see Fig. 2) with an average temperature of 21°C.

Age and gender distributions of all participants are listed in Table 1, Table 2.

**Table 1:** Age and gender distribution of participants having taken part in the project during the Days of Science and Technology 2016.

Age range	Number of men	Number of women	Total number of participants
0–10	12	8	20
11–20	27	40	67
21–30	21	15	36
31–40	12	19	31
41–50	12	27	39
51–60	3	10	13
61–70	5	7	12
71–80	5	7	12
81–90	1	3	4
91–100	0	0	0
100+	0	0	0
Sum	98	136	234

**Table 2:** Age and gender distribution of all participants having taken part in the project as the members of Mensa Czech Republic.

Age range	Number of men	Number of women	Total number of participants
0–10	1	0	1
11–20	5	6	11
21–30	4	6	10
31–40	5	6	11
41–50	9	2	11
51–60	5	3	8
61–70	1	0	1
71–80	0	0	0
81–90	0	0	0
91–100	0	0	0
100+	0	0	0
Sum	30	23	53

### 2.2 Data collection procedure

Prior to measurements all participants were familiarized with the goal of the project, overall experimental procedure and related legal conditions. Then they were registered into a software application for rapid collection, storage, processing and visualization of heterogeneous health related data (described in [3]), signed the informed consent and filled in a short motivational questionnaire (described in more detail in Section 2.3). Immediately after that they took part in individual measurements organized at nine physical sites (the number of physical measurement sites was limited for the participants from Mensa Czech Republic). Each physical site was equipped with appropriate hardware and software tools related to the type of measurement and served at least by one human expert who also provided the participant with the information about the site measurement. The last physical site, the information desk, served both for the registration of the participants and provision of measurements results. It was served by three people.

**Fig. 3:**
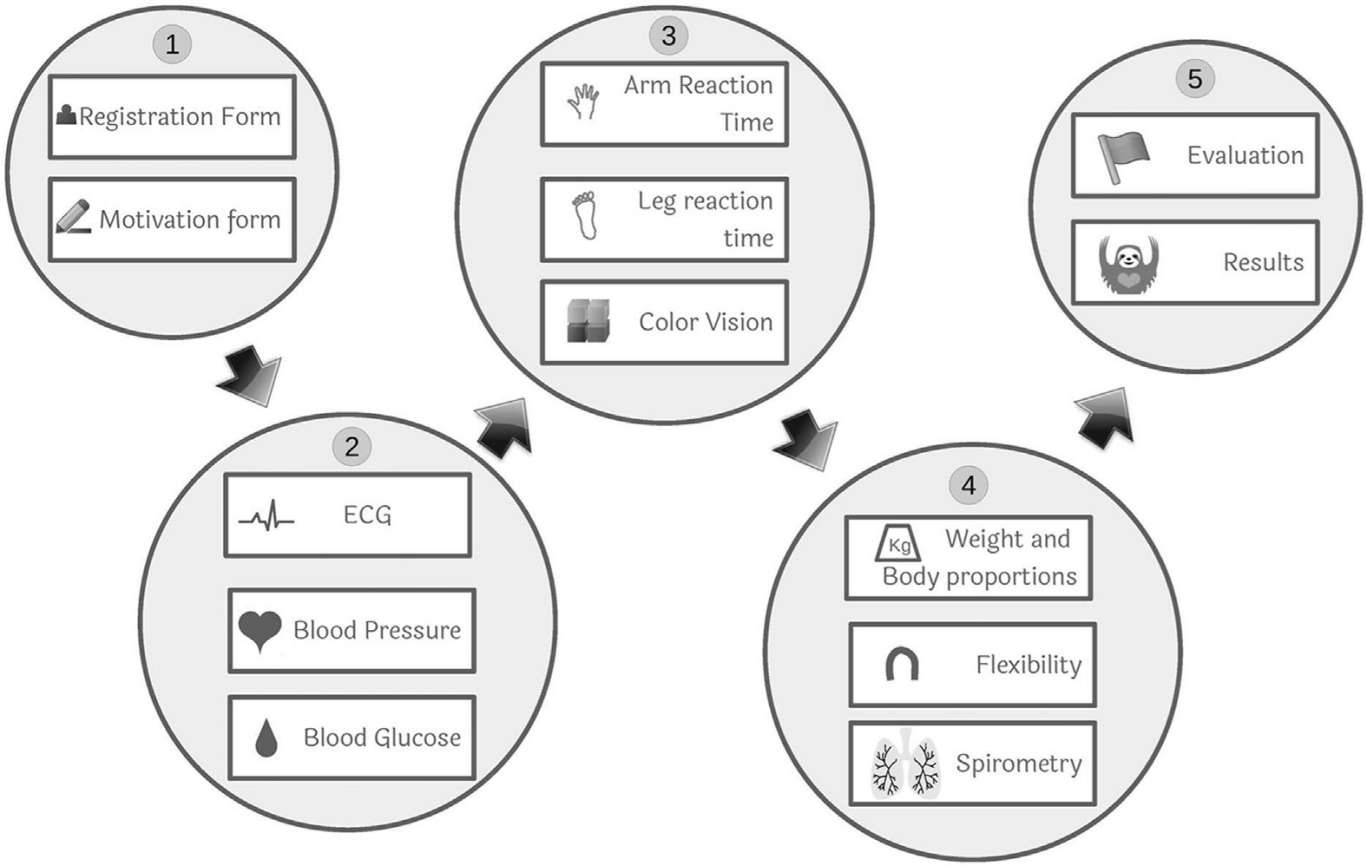
Schema of measurement sites and recommended route between them during the Days of Science and Technology 2016.

Although there was a recommended route between individual measurement sites, in fact the participants could circle them in any order (see the schema of measurement sites and the recommended route in Fig. 3). They were also not required to complete all the measurements and could have interrupted the measurement cycle at any time. Only in the best case they visited all the measurement sites and filled in all questions in the questionnaire. The complete data collection procedure took approximately 15 minutes.

When a single measurement was completed, the obtained data were inserted via a user interface into a software application. When the participant finished his/her last measurement, he/she was provided with the results (measured values) from all the visited measurement sites organized on one A4 page.

### 2.3 Motivational questionnaire

After registering and signing the informed consent each participant proceeded to fill in a motivational questionnaire containing a set of 13 single choice questions to provide a basic overview of participant's current lifestyle and health condition. The following questions were asked:

**Q1: Do you exercise regularly?**

1. Yes
2. No

**Q2:** If not. **Would you like to exercise regularly?**

1. Yes
2. No

**Q3:** If yes. **Do you have friends with whom you could exercise?**

1. Yes
2. No

**Q4: Do you eat regularly?**

1. Yes
2. No

**Q5: Do you drink enough water during the day? (2–3 liters per day)**

1. Yes
2. No

**Q6: Do you eat healthily? eg. poultry, fish, fruits, vegetables, water, etc.**

1. Yes
2. No

**Q7: Do you use any dietary supplements? e.g. vitamins, supplements for joints or bones, etc.**

1. Yes
2. No

**Q8: Do you smoke?**

1. No
2. Yes
3. Occasionally

**Q9:** If yes. **How many cigarettes do you smoke?**

1. Up to 10 cigarettes per day
2. Up to 10 cigarettes per week
3. Up to 20 cigarettes per day
4. Up to 10 cigarettes per month
5. 20 or more cigarettes per day

**Q10: How often do you drink alcoholic beverages?**

1. Every day
2. Once per week
3. Multiple times per week
4. Occasionally
5. I don't drink any alcohol

**Q11: Do you undergo regular medical examinations?**

1. Yes
2. No

**Q12: Do you have a girlfriend/boyfriend or husband/wife?**

1. Yes
2. No

**Q13: Do you indulge yourself with proper rest and relaxation? e.g. massages, wellness, proper rest (minimum of 6 hours per day),** …

1. Yes
2. No

### 2.4 Measurement sites

The number of measurements sites was different for the participants visiting the Days of Science and Technology 2016 and the participants from Mensa Czech Republic visiting the neuroinformatics laboratory at the University of West Bohemia. The restriction of measurement sites for the participants from Mensa Czech Republic (information desk, hands reaction time, legs reaction time, and color vision sites were available for them) was primarily caused by the limited time they had during their visit in the laboratory and their interest in other kinds of measurements related to brain functioning.

#### 2.4.1 Hands reaction times

The measurement site was focused on the measurement of participant's hands reaction times to outside visual stimuli.

**Fig. 4:**
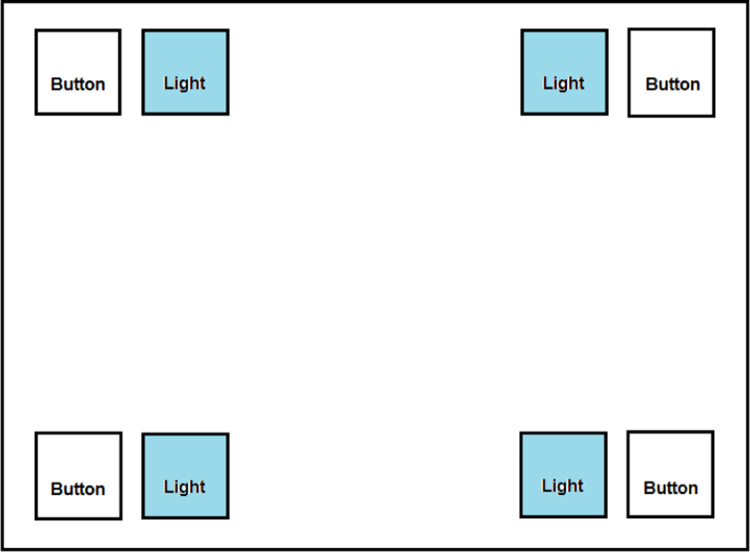
Cognitive research device for measuring hands reaction times. Only one LED panel could be active at a time.

A custom cognitive research device consisting of a wooden desk with four buttons and LED panels placed in a square formation (as is shown in Fig. 4) and related hardware and embedded control software for generation of visual stimuli (lighting up the LED panels) and recording the participant's responses [1] was used. The task of the participant was to press a button near the LED diode panel turned on by right or left hand as quickly as possible. Only one LED panel could have been active at a time. The order of lighting up the LED panels was random and controlled by embedded software. In total the participant completed 16 trials where he/he pressed one of the four buttons placed on the wooden plate according to the LED panel turned on.

The results given to the participant contained the following values (all these values were computed by the embedded control software and manually inputted by the site experimenter to the software application for rapid collection of health related data):

• Average hands reaction time [ms] – calculated from 16 trials when the participant pressed one of the four buttons placed on the wooden plate according to the LED panel turned on,
• Number of missed reactions – a missed reaction was considered when no button was pressed within the time limit one of the LED panels was turned on,
• Number of incorrect reactions – an incorrect reaction was considered when a wrong button was pressed within the time limit one of the LED panels was turned on.

#### 2.4.2 Legs reaction times

**Fig. 5:**
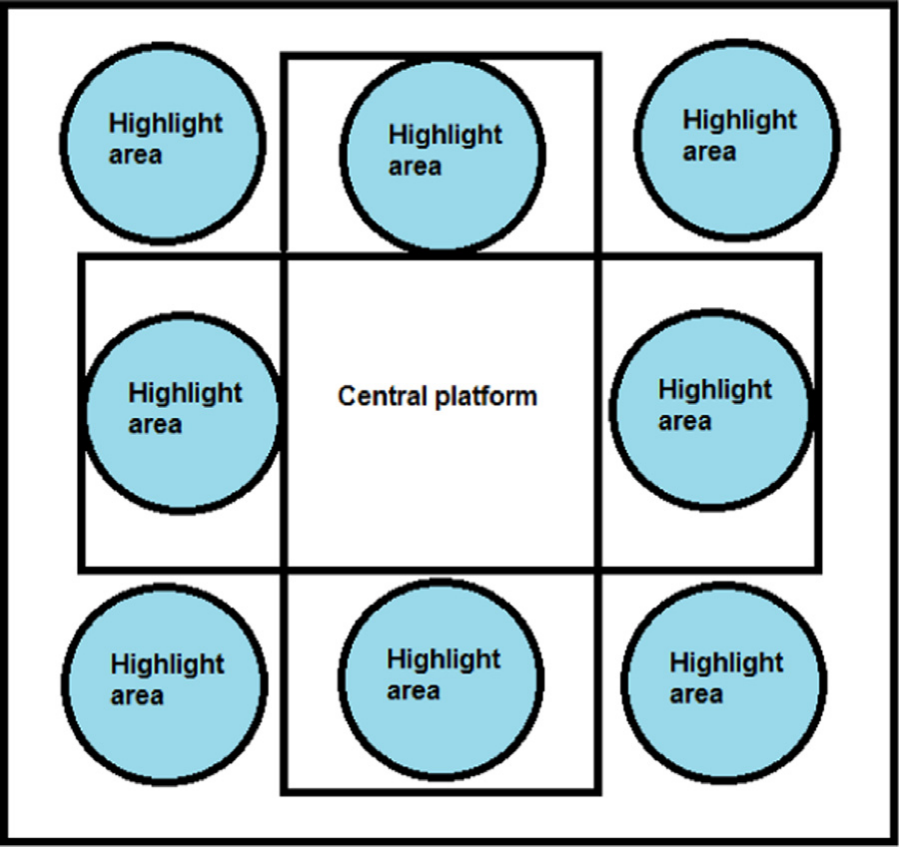
Impact dance pad for measuring legs reaction times. Only one area could be active at a time.

The next measurement site was focused on the measurement of the legs reaction time using an impact dance pad (see Fig. 5). This dance pad was divided into nine areas (central area serving as a base point and eight side areas serving as places capturing touches of the participant's leg) and connected to a laptop where these areas were represented by corresponding patterns that had been randomly highlighted. Only one area could have been active at a time. The task of the participant was to stand in the central part of the dance pad, step aside once the corresponding pattern on the laptop was highlighted and return quickly back to the central part of the dancing pad [2]. In total the participant completed 16 trials where he/she stepped aside and return back to the central position.

The results given to the participant contained the following values (all these values were computed by the custom application and manually inputted by the site experimenter to the software application for rapid collection of health related data):

• Average legs reaction time [ms] – calculated from 16 trials when the participant touched one of eight areas on the impact dance pad by his/her leg,
• Standard deviation [ms] – calculated from all trials,
• Best reaction time [ms] – the shortest reaction time achieved on the impact dance pad (limited by 2000ms interval),
• Worst reaction time [ms] – the longest reaction time achieved on the impact dance pad (limited by 2000ms interval).

#### 2.4.3 Color vision

The third measurement site was focused on the measurement of color vision. The participant was tested using a total of eight pseudochromatic pictures. His/her task was to recognize the number hidden in these pictures.

The results given to the participant contained the following values (all these values were inputted manually by the site experimenter to the software application for rapid collection of health related data):

• List of incorrectly recognized pictures.

#### 2.4.4 Spirometry

The fourth measurement site was focused on the measurement of lung capacity, forced expiratory volume, and expiratory flow of the participant. All measurements were performed using the SP10W spirometer.

The results given to the participant contained the following values (all these values were inputted manually by the site experimenter to the software application for rapid collection of health related data):

• Forced vital capacity (FVC) [l] – the amount of air the participant forcibly expelled from the lungs after taking the deepest breath possible,
• Forced expiratory volume in the 1st second (FEV1) [l] – the amount of air the participant expelled during a forced breath and measured during the first second,
• Peak expiratory flow (PEF) [l/s] – the maximum speed of participant's expiration.

#### 2.4.5 Electrocardiography (ECG)

The fifth measurement site was focused on the electrocardiography measurements. It included heart rate (HR) together with measurement of the ST segment and QRS interval. QRS representing ventricle depolarization was measured from the start of the Q wave to the end of the S wave. The ST segment was measured from the end of the S wave, J point, to the start of the T wave. All measurements were performed using the ReadMyHeart Handheld ECG device.

The results given to the participant contained the following values (all these values were inputted manually by the site experimenter to the software application for rapid collection of health related data):

• Heart rate (HR) [puls/min],
• ST Segment [mm] – the length of the ST segment that represents the interval between ventricular depolarization and repolarization,
• QRS Interval [s] – duration of the QRS complex that represents ventricle depolarization.

#### 2.4.6 Blood pressure

The sixth measurement site was focused on the measurement of blood pressure in a traditional way as a systolic and diastolic blood pressure. This measurement was completed by the measurement of heart rate, here denoted as puls. All measurements were performed using the Omron M6 Comfort IT device.

The results given to the participant contained the following values (all these values were inputted manually by the site experimenter to the software application for rapid collection of health related data):

• Systolic blood pressure [mmHg],
• Diastolic blood pressure [mmHg],
• Puls [puls/min].

#### 2.4.7 Blood glucose

The seventh measurement site was focused on the measurement of glucose concentration in blood. All measurements were performed using the FORA Diamond Mini blood glucose monitoring system.

The result given to the participant contained the following value (this value was inputted manually by the site experimenter to the software application for rapid collection of health related data):

• Glucose [mmol/l] - concentration of glucose in blood.

#### 2.4.8 Body proportions

The eight section was focused on the measurement of body proportions, the participant's height was measured manually, weight, body mass index (BMI), and concentration of muscle-mass, water and fat in participant's body was measured and calculated the Medisana BS 440 Connect device.

The results given to the participant contained the following values (these values were inputted manually by the site experimenter to the software application for rapid collection of health related data):

• Height [cm] – participant's height,
• Weight [kg] – participant's weight,
• Body Mass Index (BMI),
• Muscle-Mass [%] – concentration of muscle-mass in human body,
• Water [%] – concentration of water in human body,
• Fat [%] – concentration of fat in human body.

#### 2.4.9 Flexibility

The ninth section was focused on measurement of human body flexibility that was measured using a 13 cm high portable podium. The participant standing at the podium was asked to touch his/her own feet. Not being able to do it, the result was as a negative number, On the other hand, when the participant managed to bend even more, the result was a positive number.

The result given to the participant contained the following value (this value was inputted manually by the site experimenter to the software application for rapid collection of health related data):

• Flexibility [cm] – difference between position of fingers and foot during deep forward bend (limited by +13 cm podium height).

### 2.5 Used hardware

The following table (Table 3) summarizes devices used during the measurements.

**Table 3:** Hardware devices used at individual measurement sites.

Measurement site	Device name
Body proportions	Medisana BS 440 Connect
Electrocardiography	ReadMyHeart Handheld ECG
Blood pressure	Omron M6 Comfort IT
Blood sugar	FORA Diamond Mini
Spirometry	SP10W
Hand reaction time	Device for cognitive research [1]
Leg reaction time	Impact Dance Pad [2]
Flexibility	Podium and ruler
Color vision	Pseudoisochromatic pictures

### 2.6 Data collections

The data collected during the Days of Science and Technology 2016 are available in Table 6 (hands and legs reaction times), Table 7 (color vision and spirometry data), Table 8 (ECG, blood pressure and blood glucose data) and Table 9 (body proportions and flexibility data). The data collected in the neuroinformatics laboratory from the members of Mensa Czech Republic are available in Table 11 (hands and legs reaction times). The questionnaire data collected during the Days of Science and Technology 2016 are available in Table 10. The questionnaire data collected in the neuroinformatics laboratory are available in Table 12.

### 2.7 Preliminary statistical analysis

#### 2.7.1 Description and processing of datasets

Each record in the data collection corresponds to one participant. The first step of the preliminary statistical analysis was to distribute the obtained data into three categories. The first category *Basic data* contains the following information about each participant:

1. the group the participant belongs to (0 – the member of the Mensa Czech Republic, 1 – the person visiting the Days of Science and Technology 2016),
2. gender (0 male, 1 – female),
3. age.

The second category *Measured data* includes the subcategories that are related to the data obtained from individual measurement sites (in case of pseudochromatic pictures the value 0/1 means that the participant did not recognize/recognized the hidden number):

1. average hands reaction time [ms],
2. number of missed hands reactions,
3. number of incorrect hands reactions,
4. average legs reaction time [ms],
5. standard legs deviation,
6. best legs reaction time [ms],
7. worst legs reaction time [ms],
8. pseudochromatic picture 1 [0/1],
9. pseudochromatic picture 2 [0/1],
10. pseudochromatic picture 3 [0/1],
11. pseudochromatic picture 4 [0/1],
12. pseudochromatic picture 5 [0/1],
13. pseudochromatic picture 6 [0/1],
14. pseudochromatic picture 7 [0/1],
15. pseudochromatic picture 8 [0/1],
16. systolic pressure [mmHg],
17. diastolic pressure [mmHg],
18. puls [puls/min],
19. heart rate (HR) [puls/min],
20. ST segment [mm],
21. QRS interval [s],
22. glucose [mmol/l],
23. forced vital capacity [l],
24. forced expiratory volume in the 1st second [l],
25. peak expiratory flow [l/s],
26. height [cm],
27. weight [kg],
28. BMI,
29. muscle mass [%],
30. water [%],
31. fat [%],
32. flexibility [cm].

The last category *Questionnaire data* includes the data obtained from the questionnaires completed by the participants. These data are divided into the following subcategories:

1. Sport – the participant
  – 0 - does not do any sport + does not want to do any sport,
  – 1 - does not do any sport and wants to do some sport,
  – 2 - does some sport, but has no friends to do some sport with,
  – 3 - does some sport and has friends to do some sport with.
2. Food – the participant
  – 0 - eats irregularly and unhealthily,
  – 1 - eats irregularly and healthily,
  – 2 - eats regularly, but unhealthily,
  – 3 - eats irregularly and unhealthily.
3. Drinking habits – the participant
  – 0 - drinks enough water,
  – 1 - does not drink enough water.
4. Supplements – the participant
  – 0 - does not use any supplements,
  – 1 - uses supplements.
5. Smoking – the participant
  – 0 - does not smoke,
  – 1 - smokes up to 10 cigarettes per month,
  – 2 - smokes up to 10 cigarettes per week,
  – 3 - smokes up to 10 cigarettes per day,
  – 4 - smokes up to 20 cigarettes per day,
  – 5 - smokes 20 or more cigarettes per day.
6. Alcoholic beverages – the participant
  – 0 - does not drink any alcoholic beverages,
  – 1 - drinks alcoholic beverages occasionally,
  – 2 - drinks alcoholic beverages once a week,
  – 3 - drinks alcoholic beverages several times per week.
7. Medical checks – the participant
  – 0 - undergoes medical checks periodically,
  – 1 - undergoes medical checks irregularly.
8. Partner – the participant
  – 0 - does not have a girlfriend/boyfriend or spouse,
  – 1 - has a girlfriend/boyfriend or spouse.
9. Rest and relaxation – the participant
  – 0/1 - does not/does rest and relax,
  – 0/1 - does not/does rest and relax.

The dataset is partly inconsistent because not the whole set of health related data was obtained from each participant. Moreover, the members of Mensa Czech Republic did not participated in all measurements to obtain the whole set of health related data. The motivational questionnaire was also not filled fully in all cases. Since unfilled or inaccurate data can influence the variability of the dataset, the statistical methods that can cope with expected errors were used. The significance level of 0.05 was used for all tests. All statistical methods were performed in MATLAB.

#### 2.7.2 Basic statistical characteristics of dataset

**Fig. 6:**
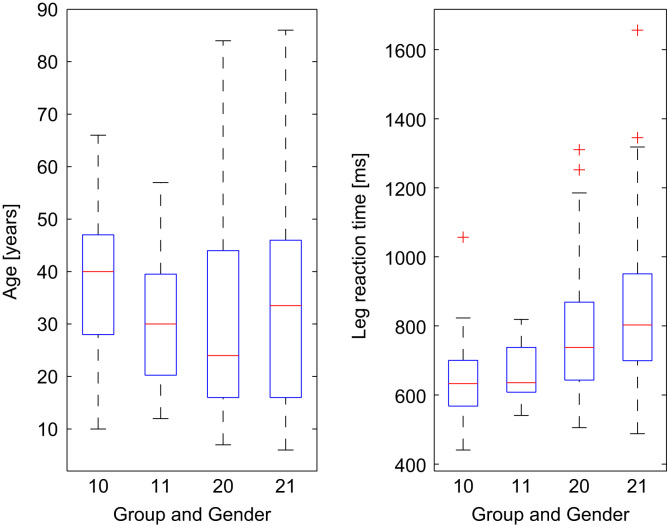
Left box plot – Age of participants (from left males from Mensa Czech Republic (10), females from Mensa Czech Republic (11), males visiting the Days of Science and Technology 2016 (21) and females visiting the Days of Science and Technology 2016 (22) ). Right box plot – legs reaction time (in the same order as in the left box plot).

**Fig. 7:**
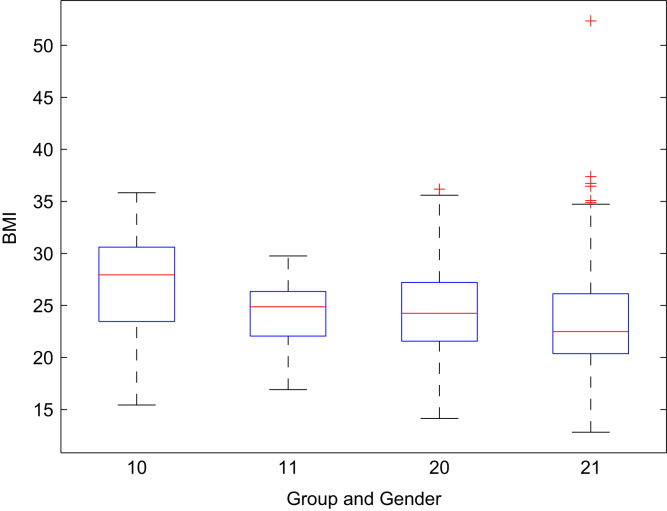
Box plot – BMI of participants (from left males from Mensa Czech Republic, females from Mensa Czech Republic, males visiting the Days of Science and Technology 2016 and females visiting the Days of Science and Technology 2016).

Box plot graphs used to visualize the basic statistical characteristics of the data were created separately for the members of Mensa Czech Republic and for the visitors of the Days of Science and Technology 2016. Fig. 6, Fig. 7 show the box plot graphs depicting the range of participants' age, legs reaction times and BMI.

#### 2.7.3 Dependency relationships of measured and questionnaire data

To find dependencies between the subcategories of measured and questionnaire data linear regression in the form of general matrix Y=βX+ϵ was used, where *Y* is the vector of response variables (measured data) and *X* is the vector of explanatory variables (questionnaire data), ϵ is a random component of the linear model. To construct linear regression models the following methods were used.

The first method was based on the gradual selection of questionnaire data subcategories. On the base of significance coefficients and p-values it was decided which questionnaire data subcategory significantly affects the selected subcategory of the measured data.

The questionnaire data were regarded as one package in the second method. The multivariate regression analysis for all subcategories of the questionnaire data processed simultaneously was implemented.

In the third method the stepwise regression was used. This method is useful in case of large amount of explanatory regressors (the questionnaire data subcategories in our case). Gradual draining of individual explanatory variables, for which $H_{0}:\beta_{i}=0$ cannot be dismissed, simplifies the regression model and identification of statistically significant explanatory regressors.

The stepwise regression was chosen as the most suitable method for the dataset. The results of this regression are summarized in Table 4, Table 5 (value 1 corresponds to the fact that the explanatory variable significantly affects the response variable, 0 corresponds to the independence of two variables).

**Table 4:** Statistical data – part 1.

	Sport	Food	Drinking regime	Supplements	Smoking
Average hands reaction time [ms]	1	0	0	0	0
Number of missed hands reactions	0	0	0	0	0
Number of incorrect hands reactions	0	0	0	0	0
Average legs reaction time [ms]	0	0	0	0	0
Standard legs deviation	0	0	0	0	0
Best legs reaction time [ms]	0	0	0	0	0
Worst legs reaction time [ms]	0	0	0	0	0
Pseudochromatic picture 1	0	0	0	0	0
Pseudochromatic picture 2	0	0	0	0	0
Pseudochromatic picture 3	0	0	0	0	0
Pseudochromatic picture 4	0	0	0	0	0
Pseudochromatic picture 5	0	0	0	0	0
Pseudochromatic picture 6	0	0	0	0	0
Pseudochromatic picture 7	0	0	0	0	0
Pseudochromatic picture 8	0	0	0	0	0
Systolic pressure [mmHg]	0	0	0	0	0
Diastolic pressure [mmHg]	1	0	0	0	0
Puls [puls/min]	0	0	0	0	0
HR [puls/min]	0	0	0	0	0
ST segment [mm]	0	0	0	0	0
QRS interval [s]	0	0	0	0	0
Glucose [mmol / l]	0	0	0	0	0
Forced vital capacity [l]	0	0	0	0	0
Forced expiratory volume in the 1st second [l]	0	0	0	0	0
Peak expiratory flow [l / s]	0	0	0	0	0
Height [cm]	0	0	0	0	0
Weight [kg]	0	1	0	0	0
BMI	0	1	0	0	0
Muscle mass [%]	0	0	0	0	0
Water [%]	0	0	0	0	0
Fat [%]	0	0	0	0	0
Flexibility [cm]	0	0	0	0	0

**Table 5:** Statistical data – part 2.

	Alcohol habits	Medical checks	Partner	Rest/relax
Average hands reaction time [ms]	0	0	0	0
Number of missed hands reactions	1	0	1	0
Number of incorrect hands reactions	0	0	0	0
Average legs reaction time [ms]	0	0	0	0
Standard legs deviation	0	0	0	0
Best legs reaction time [ms]	1	0	0	0
Worst legs reaction time [ms]	0	0	0	0
Pseudochromatic picture 1	1	0	0	0
Pseudochromatic picture 2	1	0	0	0
Pseudochromatic picture 3	1	0	0	0
Pseudochromatic picture 4	1	0	0	0
Pseudochromatic picture 5	0	0	0	0
Pseudochromatic picture 6	1	0	0	0
Pseudochromatic picture 7	0	0	0	0
Pseudochromatic picture 8	1	0	0	0
Systolic pressure [mmHg]	0	0	0	0
Diastolic pressure [mmHg]	0	0	0	0
Puls [puls/min]	0	0	0	0
HR [puls/min]	0	0	0	0
ST segment [mm]	0	0	0	0
QRS interval [s]	0	0	0	0
Glucose [mmol / l]	0	0	0	0
Forced vital capacity [l]	0	0	0	0
Forced expiratory volume in the 1st second [l]	0	0	0	0
Peak expiratory flow [l / s]	0	0	0	0
Height [cm]	0	0	0	0
Weight [kg]	0	1	0	0
BMI	0	1	0	0
Muscle mass [%]	0	0	0	0
Water [%]	0	0	0	0
Fat [%]	0	0	0	0
Flexibility [cm]	0	0	0	0

## 3 Data tables

**Table 6:** **Data collected from visitors during the Days of Science and Technology 2016**. ID - id of the participant, Sex - sex of the participant, Age - age of the participant, RT-H [ms] - hands average reaction time, MH - the number of missed buttons during hands reaction time measurement, EH - the number of wrongly pressed buttons during hands reaction time measurement, RT-L [ms] - legs average reaction time, StD-L [ms] - standard deviation between responses during legs reaction time measurement, BT-L [ms] - best (shortest) time achieved during legs reaction time measurement, WT-L [ms] - worst (longest) time achieved during legs reaction time measurement, “—” - blank data (data were not filled in).

ID	Sex	Age	RT-H[ms]	MH	EH	RT-L[ms]	StD-L[ms]	BT-L[ms]	WT-L[ms]
1	Male	24	659	7	—	540	206	456	1069
2	Female	51	986	10	—	879	109	734	1085
3	—	—	—	—	—	—	—	—	—
4	—	—	—	—	—	—	—	—	—
5	—	—	—	—	—	—	—	—	—
6	—	—	—	—	—	—	—	—	—
7	Female	82	919	10	—	1318	348	876	2000
8	—	—	—	—	—	—	—	—	—
9	—	—	—	—	—	—	—	—	—
10	—	—	—	—	—	—	—	—	—
11	Female	34	—	—	—	832	318	584	2000
12	Male	16	562	0	1	655	200	513	1390
13	Male	16	734	3	1	624	64	503	728
14	Male	17	608	7	1	543	216	620	820
15	Female	14	913	6	1	797	172	619	1414
16	—	—	—	—	—	—	—	—	—
17	—	—	—	—	—	—	—	—	—
18	—	—	—	—	—	—	—	—	—
19	—	—	—	—	—	—	—	—	—
20	Male	12	963	3	3	728	131	489	991
21	Male	12	959	5	1	783	118	545	1085
22	Male	15	999	5	0	760	83	561	909
23	—	—	—	—	—	—	—	—	—
24	Female	45	954	4	—	713	104	538	920
25	—	—	—	—	—	—	—	—	—
26	—	—	—	—	—	—	—	—	—
27	—	—	—	—	—	—	—	—	—
28	Female	13	807	7	1	738	113	527	958
29	Female	12	892	2	0	740	290	514	1538
30	Female	12	922	1	1	610	197	459	943
31	—	—	—	—	—	—	—	—	—
32	—	—	—	—	—	—	—	—	—
33	Female	29	1016	3	0	878	631	553	2000
34	Female	17	1049	1	0	652	73	537	763
35	Male	17	815	3	0	718	187	502	1065
36	Female	16	668	2	0	556	189	507	1002
37	Female	16	932	1	3	723	270	542	1237
38	Female	17	744	0	1	689	86	574	843
39	Male	26	873	3	1	657	101	543	935
40	Male	16	824	0	2	673	57	573	781
41	Female	46	1097	3	0	925	199	735	1542
42	—	—	—	—	—	—	—	—	—
43	Male	18	666	0	0	632	224	482	1041
44	—	—	—	—	—	—	—	—	—
45	—	—	—	—	—	—	—	—	—
46	—	—	—	—	—	—	—	—	—
47	Male	16	843	2	0	854	353	609	1920
48	Male	16	582	0	0	593	47	510	696
49	—	—	—	—	—	—	—	—	—
50	—	—	—	—	—	—	—	—	—
51	—	—	—	—	—	—	—	—	—
52	Male	14	—	—	—	1185	268	818	2000
53	—	—	—	—	—	—	—	—	—
54	—	—	—	—	—	—	—	—	—
55	—	—	—	—	—	—	—	—	—
56	—	—	—	—	—	—	—	—	—
57	—	—	—	—	—	—	—	—	—
58	—	—	—	—	—	—	—	—	—
59	Female	27	966	4	0	960	374	659	1954
60	—	—	—	—	—	—	—	—	—
61	—	—	—	—	—	—	—	—	—
62	Female	70	0	15	0	981	486	681	2000
63	—	—	—	—	—	—	—	—	—
64	—	—	—	—	—	—	—	—	—
65	—	—	—	—	—	—	—	—	—
66	Male	55	1083	11	0	797	105	572	1023
67	—	—	—	—	—	—	—	—	—
68	—	—	—	—	—	—	—	—	—
69	Male	17	902	6	0	720	322	507	1926
70	Male	17	961	9	0	545	224	480	1215
71	Female	55	750	6	1	622	68	509	767
72	Female	42	831	7	1	701	279	561	1513
73	Female	14	835	9	0	589	327	440	1834
74	Female	13	1010	8	1	685	203	597	1044
75	Female	17	885	5	1	778	425	511	2000
76	Female	54	770	0	1	721	238	521	1052
77	—	—	—	—	—	—	—	—	—
78	Female	70	598	11	0	867	237	826	1116
79	Male	71	800	12	0	650	192	569	902
80	Male	12	—	—	—	915	197	608	1194
81	—	—	—	—	—	—	—	—	—
82	Male	14	598	5	2	724	114	569	1078
83	Female	14	558	8	0	705	80	563	854
84	—	—	—	—	—	—	—	—	—
85	—	—	—	—	—	—	—	—	—
86	Female	19	747	9	0	1041	234	700	1514
87	Female	53	954	8	1	655	185	1	874
88	Female	83	1116	10	0	1248	216	1659	845
89	Male	64	988	9	1	739	215	674	1037
90	Male	11	738	6	0	854	122	627	1143
91	Female	40	789	8	1	660	188	601	887
92	—	—	—	—	—	—	—	—	—
93	Male	9	524	3	0	734	246	506	1367
94	Male	9	666	4	0	924	138	700	1203
95	Female	19	614	2	0	689	45	586	755
96	Male	62	889	5	1	781	234	723	1154
97	Female	33	—	—	—	—	—	—	—
98	Female	55	988	8	0	855	376	697	2000
99	Male	45	659	2	2	727	95	590	955
100	Male	24	1018	7	0	687	351	450	1980
101	Female	58	950	8	0	1046	478	594	2000
102	Male	60	1043	7	1	861	441	466	2000
103	Female	14	597	5	0	858	281	565	1762
104	Female	14	813	8	1	735	266	554	1709
105	Female	12	1059	10	1	863	125	678	1158
106	Female	14	1035	6	0	1036	385	669	2000
107	Female	15	798	3	0	—	—	—	—
108	Male	17	583	5	0	715	319	516	1556
109	Female	19	727	10	1	635	150	533	1187
110	Female	63	756	5	3	900	327	569	1848
111	—	—	—	—	—	—	—	—	—
112	Female	31	774	3	0	956	410	630	2000
113	Male	27	554	2	0	763	381	519	1971
114	Female	41	544	12	0	695	201	450	1369
115	Female	9	934	10	0	775	143	638	1276
116	Female	19	—	—	—	—	—	—	—
117	Female	47	660	11	2	686	93	526	889
118	Female	6	1004	12	1	—	—	—	—
119	Female	46	1126	11	1	—	—	—	—
120	Female	43	488	3	2	618	180	435	484
121	Male	7	961	6	0	—	—	—	—
122	Male	10	893	5	0	950	373	539	2000
123	Male	7	—	—	—	—	—	—	—
124	Male	27	622	7	0	646	90	504	841
125	Female	44	752	4	0	947	374	702	1780
126	Female	15	571	4	0	611	255	495	1341
127	Male	45	698	4	0	800	450	615	2000
128	Female	13	743	6	2	847	339	547	2000
129	Female	17	823	8	1	940	252	705	1738
130	Female	17	607	4	0	582	149	447	1114
131	Male	23	421	4	0	624	63	544	785
132	Female	24	701	6	0	781	174	591	1238
133	Male	38	968	2	0	830	100	648	1023
134	Female	42	752	5	1	782	186	606	1405
135	Female	73	878	3	2	1313	466	884	2000
136	Female	38	556	4	0	693	261	600	1380
137	Female	6	1185	10	0	1656	206	1244	2000
138	Male	26	817	2	0	814	368	702	2000
139	Female	25	659	2	0	873	577	665	2000
140	Female	40	—	—	—	—	—	—	—
141	Male	23	518	4	2	577	261	443	1394
142	Female	20	546	6	1	631	83	462	767
143	Male	23	416	4	0	506	40	445	605
144	Female	27	544	2	1	651	100	498	918
145	Male	28	642	6	1	671	145	464	1133
146	Male	69	975	—	0	1134	187	738	1390
147	Male	18	820	—	0	630	238	582	1324
148	Female	20	845	—	0	803	119	597	1068
149	Female	25	664	—	1	837	299	603	1915
150	Male	25	862	—	0	628	49	555	712
151	Female	45	957	—	0	745	175	536	1095
152	Female	55	883	—	0	854	173	659	1318
153	Male	13	749	—	1	758	214	552	1507
154	Male	71	888	—	1	976	298	716	2000
155	Male	61	1013	—	0	1017	143	762	1283
156	—	—	—	—	—	—	—	—	—
157	Male	73	1045	—	3	1018	386	818	2000
158	Male	38	803	—	1	834	132	610	1167
159	Male	10	972	—	0	869	491	582	2000
160	Female	11	951	—	0	945	158	759	1292
161	Female	14	993	—	0	688	150	502	1501
162	Female	8	1077	—	1	1345	277	981	1968
163	Male	17	920	4	0	924	296	645	2000
164	—	—	—	—	—	—	—	—	—
165	Female	27	765	—	0	1106	520	678	2000
166	Male	49	1099	—	0	918	374	601	2000
167	Female	45	911	—	1	778	278	690	1515
168	Male	15	1166	1	1	772	247	610	1683
169	Female	38	—	—	—	—	—	—	—
170	Female	31	961	—	0	814	142	639	1149
171	Female	38	968	—	0	739	170	519	1199
172	Male	30	1012	—	0	718	95	573	889
173	Female	14	949	—	1	610	82	467	770
174	Female	10	959	—	2	911	375	683	2000
175	Female	7	1006	—	0	1190	151	1006	1704
176	—	—	—	—	—	—	—	—	—
177	—	—	—	—	—	—	—	—	—
178	Male	13	809	—	0	636	345	430	1610
179	Female	41	780	—	0	1055	162	788	1348
180	Male	14	1002	—	0	735	137	535	1038
181	Female	12	905	2	2	556	164	490	803
182	Female	45	721	0	0	673	190	598	855
183	Male	40	967	—	1	605	112	478	840
184	Male	8	941	—	1	932	179	762	1545
185	Male	50	783	—	0	999	306	699	2000
186	Female	43	910	1	0	604	47	505	676
187	Female	51	781	—	1	596	167	492	746
188	—	—	—	—	—	—	—	—	—
189	Female	52	1078	—	0	709	254	485	1530
190	Male	24	777	—	1	595	60	503	729
191	Female	15	809	9	0	731	110	575	1045
192	Female	23	866	—	0	871	434	594	2000
193	Male	73	1247	6	0	1310	305	1055	2000
194	Male	50	837	—	2	564	240	440	1295
195	Male	40	1360	—	—	717	85	504	928
196	Male	8	1006	—	—	1111	341	813	2000
197	Female	72	1102	13	1	1219	244	930	1752
198	Female	40	918	—	—	488	133	453	618
199	Male	43	943	10	0	682	165	505	1198
200	—	—	—	—	—	—	—	—	—
201	Male	27	911	—	—	536	151	495	717
202	Female	15	1098	—	0	1019	341	805	1743
203	Female	80	—	—	—	—	—	—	—
204	Male	84	—	—	—	—	—	—	—
205	Female	40	—	—	—	788	106	545	1038
206	Male	9	950	—	1	1072	478	622	2000
207	Female	73	1067	—	0	1209	345	827	2000
208	Male	23	836	—	—	631	359	455	2000
209	Female	36	972	—	0	760	331	555	2000
210	Female	26	822	—	1	955	267	592	1649
211	Male	32	925	—	1	818	203	554	1432
212	Male	52	739	—	0	639	381	472	1872
213	Female	47	1150	—	1	1103	262	880	2000
214	Female	31	956	—	0	699	91	593	985
215	Female	72	887	2	0	1115	445	930	2000
216	—	—	—	—	—	—	—	—	—
217	—	—	—	—	—	—	—	—	—
218	Female	80	1006	3	2	972	118	807	1323
219	Male	10	920	—	0	1006	428	599	2000
220	Male	40	664	0	0	747	221	535	1422
221	Female	27	1062	3	1	1188	548	748	2000
222	Male	44	827	2	1	728	291	644	930
223	Male	27	994	1	1	1252	316	1003	2000
224	Male	37	877	2	3	988	121	822	1325
225	—	—	—	—	—	—	—	—	—
226	Female	49	903	1	1	777	277	580	1171
227	Male	49	979	1	1	887	353	612	2000
228	Female	41	820	0	0	756	110	609	1076
229	Female	8	958	1	0	1022	513	821	2000
230	Female	62	873	1	0	773	114	641	1075
231	Female	26	972	3	0	738	339	544	2000
232	Female	63	880	2	1	890	139	705	1163
233	Female	46	724	2	1	952	390	619	2000
234	Male	16	666	0	0	643	356	453	2000
235	Female	13	749	0	2	719	388	531	2000
236	Male	49	767	1	0	—	—	—	—
237	Male	15	946	2	0	847	260	584	1520
238	Female	71	874	1	1	847	260	583	1520
239	Female	65	871	2	1	1204	285	977	2000
240	Female	16	717	1	0	877	316	567	2000
241	Female	15	691	0	0	722	106	547	891
242	Male	69	1194	5	5	930	398	739	2000
243	Female	24	865	2	0	—	—	—	—
244	Male	23	628	0	0	—	—	—	—
245	Female	34	657	4	0	805	259	691	1297
246	Male	48	797	7	0	859	357	616	1754
247	Female	61	1153	4	0	1268	423	765	2000
248	Female	60	859	0	0	920	290	704	2000
249	Male	36	919	0	0	637	66	540	776
250	Female	25	921	2	1	669	373	457	1942
251	Male	24	919	1	1	—	—	—	—
252	Male	23	838	4	0	—	—	—	—
253	Female	50	1015	6	0	998	261	742	1948
254	Male	37	808	2	0	754	432	494	2000
255	—	—	—	—	—	—	—	—	—
256	Female	41	903	3	0	715	218	606	1067
257	Female	31	963	3	0	—	—	—	—
258	Male	9	962	2	1	916	383	702	1880
259	Female	39	1097	2	0	619	101	480	859
260	Female	48	1059	1	1	809	78	687	974
261	Female	42	1216	4	0	815	243	724	1066
262	Male	38	846	3	0	—	—	—	—
263	Female	9	976	6	0	—	—	—	—
264	Female	11	735	2	0	691	181	600	795
265	Female	45	805	1	0	1033	561	567	2000
266	Male	10	773	1	1	699	97	549	882
267	Female	47	865	0	0	832	345	562	2000
268	Female	40	742	6	0	778	180	551	1330
269	Female	18	905	2	0	826	334	545	2000
270	—	—	—	—	—	—	—	—	—
271	—	—	—	—	—	—	—	—	—
272	Female	43	844	5	0	803	243	776	1115
273	Female	14	954	4	0	970	526	630	2000
274	Female	29	955	2	0	906	134	701	1193
275	—	—	—	—	—	—	—	—	—
276	Male	49	876	1	1	784	353	508	1616
277	Female	16	701	1	0	743	458	474	2000
278	Male	48	728	1	1	737	340	518	2000
279	Female	42	1045	9	0	1258	302	931	2000
280	Female	46	781	2	0	633	49	549	713
281	Male	32	847	4	0	581	168	483	769
282	Male	75	978	3	0	1174	409	744	2000
283	Female	86	1158	11	1	1317	309	950	2000
284	—	—	—	—	—	—	—	—	—
285	Male	35	825	1	0	583	184	631	1407
286	Female	26	709	2	0	780	107	585	1037
287	Male	28	793	1	0	731	78	610	853
288	Male	22	696	1	0	512	146	458	720
289	Female	40	926	5	1	—	—	—	—
290	Female	37	991	6	0	—	—	—	—
291	Male	17	—	—	—	780	369	684	1601
292	—	4	—	—	—	1605	230	1220	2000
293	—	8	—	—	—	1140	225	826	1682

**Table 7:** **Data collected from visitors during the Days of Science and Technology 2016** ID - id of the participant, CV1 - CV8 - indexes of pseudoisochromatic pictures (1 - right answer, 0 - wrong answer, FVC[l] - forced vital capacity in liters, FEV1[l] - forced expiratory volume in the 1st second in liters, PEF [l/s] - peak expiratory flow in liters per second, “—” - blank data (data were not filled in).

ID	CV1	CV2	CV3	CV4	CV5	CV6	CV7	CV8	FVC[l]	FEV1[l]	PEF[l/s]
1	1	1	1	1	1	1	0	1	3.88	3.05	8.94
2	—	—	—	—	—	—	—	—	2.53	2.08	2.53
3	—	—	—	—	—	—	—	—	—	—	—
4	—	—	—	—	—	—	—	—	—	—	—
5	—	—	—	—	—	—	—	—	—	—	—
6	—	—	—	—	—	—	—	—	—	—	—
7	1	1	1	1	1	0	0	1	2.1	1.84	4.26
8	—	—	—	—	—	—	—	—	—	—	—
9	—	—	—	—	—	—	—	—	—	—	—
10	—	—	—	—	—	—	—	—	—	—	—
11	1	1	1	1	1	1	0	1	3.68	3.1	5.73
12	1	1	1	1	0	1	1	1	4.98	4.16	8.46
13	1	1	1	1	0	0	0	1	3.53	2.85	5.6
14	1	1	1	1	1	1	1	1	4.4	3.64	7.78
15	1	1	1	1	0	1	0	1	3.33	2.77	3.42
16	—	—	—	—	—	—	—	—	—	—	—
17	—	—	—	—	—	—	—	—	—	—	—
18	—	—	—	—	—	—	—	—	—	—	—
19	—	—	—	—	—	—	—	—	—	—	—
20	1	1	1	1	0	1	0	1	2.07	2.04	4.47
21	1	1	1	1	0	0	1	1	2.58	2.23	4.19
22	1	1	1	1	1	1	1	1	4.67	3.98	8.18
23	—	—	—	—	—	—	—	—	—	—	—
24	1	1	1	1	1	1	0	1	3.77	3.39	5.92
25	—	—	—	—	—	—	—	—	—	—	—
26	—	—	—	—	—	—	—	—	—	—	—
27	—	—	—	—	—	—	—	—	—	—	—
28	1	1	1	1	1	1	1	1	3.19	2.72	4.9
29	1	1	1	1	0	1	0	1	2.62	2.51	15.37
30	1	1	1	1	0	0	1	1	2.03	1.75	2.78
31	—	—	—	—	—	—	—	—	—	—	—
32	—	—	—	—	—	—	—	—	—	—	—
33	1	1	1	1	1	1	0	1	3.6	3.38	5.53
34	1	1	1	1	1	1	0	1	3.63	3.43	6.43
35	1	1	1	1	1	1	0	1	10.13	2.52	9.53
36	1	1	1	1	0	1	0	1	3.24	2.96	6.27
37	1	1	1	1	1	1	1	1	3.26	1.3	1.34
38	1	1	1	1	0	1	0	1	3.35	2.85	3.72
39	1	1	1	1	0	1	1	1	4.33	3.68	7.33
40	1	1	1	1	0	0	1	1	5.51	4.76	9.33
41	1	1	1	1	1	0	0	1	4.32	3.45	5.34
42	—	—	—	—	—	—	—	—	—	—	—
43	1	1	1	1	1	1	1	1	4.56	3.65	4.52
44	—	—	—	—	—	—	—	—	—	—	—
45	—	—	—	—	—	—	—	—	—	—	—
46	—	—	—	—	—	—	—	—	—	—	—
47	1	1	1	1	0	1	0	1	3.94	3.38	5.65
48	1	1	1	1	0	1	1	1	4.9	4.9	8.24
49	—	—	—	—	—	—	—	—	—	—	—
50	—	—	—	—	—	—	—	—	—	—	—
51	—	—	—	—	—	—	—	—	—	—	—
52	—	—	—	—	—	—	—	—	—	—	—
53	—	—	—	—	—	—	—	—	—	—	—
54	—	—	—	—	—	—	—	—	—	—	—
55	—	—	—	—	—	—	—	—	—	—	—
56	—	—	—	—	—	—	—	—	—	—	—
57	—	—	—	—	—	—	—	—	—	—	—
58	—	—	—	—	—	—	—	—	—	—	—
59	1	1	1	1	1	1	1	1	3.18	2.81	4.09
60	—	—	—	—	—	—	—	—	—	—	—
61	—	—	—	—	—	—	—	—	—	—	—
62	1	1	1	1	1	1	1	1	2.75	2.31	4.21
63	—	—	—	—	—	—	—	—	—	—	—
64	—	—	—	—	—	—	—	—	—	—	—
65	—	—	—	—	—	—	—	—	—	—	—
66	1	1	1	1	1	1	1	1	4.03	3.59	10.72
67	—	—	—	—	—	—	—	—	—	—	—
68	—	—	—	—	—	—	—	—	—	—	—
69	1	1	1	1	0	1	1	1	5.07	4.37	7.68
70	1	1	1	1	1	1	1	1	3.63	3.52	7.8
71	1	1	1	1	1	1	1	1	3.16	2.61	5.81
72	1	1	1	1	1	0	1	1	3.35	2.66	6.63
73	1	1	1	1	0	1	0	1	3.52	3.16	5.02
74	1	1	1	1	1	1	1	1	3.04	2.8	4.81
75	1	1	1	1	0	1	1	1	3.73	3.5	8.03
76	1	1	1	1	1	1	1	1	2.8	2.57	6.85
77	—	—	—	—	—	—	—	—	—	—	—
78	1	1	1	1	0	0	0	1	1.71	1.45	3.99
79	1	1	1	1	1	1	0	1	2.58	2.48	6.6
80	—	—	—	—	—	—	—	—	—	—	—
81	—	—	—	—	—	—	—	—	—	—	—
82	1	1	1	1	1	1	0	1	4.49	3.53	4.27
83	1	1	1	1	1	1	1	1	3.74	2.63	2.92
84	—	—	—	—	—	—	—	—	—	—	—
85	—	—	—	—	—	—	—	—	—	—	—
86	1	1	1	1	1	1	1	1	4.44	3.46	7.5
87	1	1	1	1	1	0	1	1	2.81	2.26	6.36
88	0	1	1	1	1	1	0	1	1.74	1.49	4.98
89	1	1	1	1	0	1	0	1	3.6	3.49	9.69
90	1	1	1	1	0	1	0	1	2.43	2.13	3.54
91	1	1	1	1	1	1	1	1	3.33	3.12	5.86
92	—	—	—	—	—	—	—	—	—	—	—
93	1	1	1	1	0	0	0	1	1.71	1.27	1.64
94	1	1	1	1	0	1	0	1	2.09	1.69	3.88
95	1	1	1	1	1	1	1	1	2.96	2.67	5.97
96	1	1	1	1	1	0	0	1	4.7	3.91	8.94
97	—	—	—	—	—	—	—	—	—	—	—
98	1	1	1	1	0	1	1	1	2.43	2.33	5.34
99	1	1	1	1	0	1	1	1	3.54	3.31	6.46
100	1	1	1	1	1	1	1	1	3.34	3.29	11.35
101	1	1	1	1	1	1	1	1	3.63	3.4	5.65
102	1	1	1	1	1	0	0	1	3.75	3.36	8.24
103	1	1	1	1	0	0	0	1	3.35	3.14	5.29
104	1	1	1	1	1	1	1	1	3.07	2.57	4.13
105	1	1	1	1	1	1	0	1	2.19	2	3.05
106	1	1	1	1	0	1	0	1	2.75	1.9	2.29
107	1	1	1	1	1	1	1	1	2.37	2.27	4.39
108	1	1	1	1	1	1	1	1	5.28	4.08	7.46
109	1	1	1	1	1	1	0	1	3.5	2.34	3.05
110	1	1	1	1	0	1	0	1	3.73	3.14	7.24
111	—	—	—	—	—	—	—	—	—	—	—
112	1	1	1	1	1	1	1	1	3.72	2.96	3.6
113	1	1	1	1	1	1	1	1	3.64	3.58	11.05
114	1	1	1	1	1	0	1	1	3.94	2.23	2.66
115	1	1	1	1	0	0	0	1	2.04	1.68	2.54
116	—	—	—	—	—	—	—	—	—	—	—
117	1	1	1	1	1	1	1	1	2.51	2.36	4.7
118	1	1	1	1	0	0	0	0	1.18	1.18	3.3
119	1	1	1	1	1	0	1	1	2.75	2.36	4.44
120	1	1	1	1	1	1	1	1	3.38	2.81	8.03
121	1	1	1	1	0	0	0	1	1.29	1.22	3.32
122	—	—	—	—	—	—	—	—	2.19	2.04	3.84
123	—	—	—	—	—	—	—	—	—	—	—
124	1	1	1	1	0	1	0	1	5.67	4.26	8.4
125	1	1	1	1	1	1	1	1	2.53	2.19	6.36
126	1	1	1	1	1	1	1	1	2.66	2.42	6.43
127	1	1	1	1	1	1	1	1	4.6	3.63	9.62
128	1	1	1	1	1	1	1	1	1.95	1.7	2.1
129	1	1	1	1	0	0	0	0	2.49	2.44	4.68
130	1	1	1	1	1	1	1	1	3.02	2.9	7.2
131	1	1	1	1	0	1	0	1	4.85	4.23	9.92
132	1	1	1	1	0	1	0	1	3.35	2.26	2.99
133	1	1	1	1	1	1	1	0	4.57	3.29	7.28
134	1	1	1	1	1	1	1	1	2.39	2.31	6.89
135	1	1	1	1	1	0	1	1	1.66	1.2	2.83
136	1	1	1	1	0	1	1	1	2.84	2.55	6.7
137	0	1	1	1	1	1	1	1	1.31	0.98	1.25
138	1	1	1	1	0	1	0	1	5.56	4.98	9.06
139	1	1	1	1	0	1	1	1	3.59	2.86	3.59
140	—	—	—	—	—	—	—	—	—	—	—
141	1	1	1	1	0	1	1	1	5.61	4.65	6.96
142	1	1	1	1	1	1	1	1	4.12	3.38	4.24
143	1	1	1	1	1	1	1	1	6.33	5.51	9.69
144	1	1	1	1	1	1	1	1	3.98	3.1	4.79
145	1	1	1	1	1	1	1	1	5.43	2.6	4.23
146	1	1	1	1	0	0	0	1	3.82	3.4	9.4
147	1	1	1	1	1	1	1	1	4.25	3.86	8.46
148	1	1	1	1	1	1	1	1	2.66	2.61	6.21
149	1	1	1	1	1	1	1	1	3.11	2.8	4.55
150	1	1	1	1	1	1	1	1	4.51	4.15	10
151	1	1	1	1	1	1	1	1	2.99	2.91	6.09
152	1	1	1	1	1	1	1	1	3.44	2.94	4.85
153	1	1	1	1	1	1	0	1	2.1	2.08	6.24
154	1	1	1	1	0	0	0	1	3.4	2.87	5.92
155	1	1	1	1	1	1	0	1	4.54	4.47	10.5
156	—	—	—	—	—	—	—	—	—	—	—
157	1	1	1	1	0	1	1	1	2.26	1.18	6.4
158	1	1	1	1	1	1	0	1	3.34	3.21	9.06
159	1	1	1	1	0	1	0	1	2.09	1.7	2.54
160	1	1	1	1	0	0	0	1	1.86	1.65	2.1
161	1	1	1	1	0	0	0	1	2.38	2.02	2.8
162	1	1	1	1	0	0	0	1	1.7	1.67	3.2
163	0	1	1	1	1	1	1	1	4.01	3.53	5.34
164	—	—	—	—	—	—	—	—	—	—	—
165	0	1	1	1	1	0	0	1	2.75	2.51	6.36
166	1	1	1	1	0	1	0	1	3.55	3.52	9.33
167	1	1	1	1	0	1	1	1	2.65	2.38	4.08
168	1	1	1	1	0	1	0	1	2.94	2.84	5.25
169	1	1	1	1	0	1	0	1	—	—	—
170	1	1	1	1	0	1	1	1	2.72	2.27	5.53
171	1	1	1	1	1	1	0	1	2.43	2.38	5.75
172	1	1	1	1	1	0	1	1	5.68	3.84	10.33
173	1	1	1	1	0	0	1	1	3.29	3.16	5.73
174	1	1	1	1	0	0	0	0	2.9	2.46	3.1
175	1	1	1	1	1	1	0	1	1.6	1.55	2.85
176	—	—	—	—	—	—	—	—	—	—	—
177	—	—	—	—	—	—	—	—	—	—	—
178	1	1	1	1	1	1	0	1	2.94	2.55	3.83
179	1	1	1	1	1	1	1	1	2.99	2.36	3.09
180	1	1	1	1	1	0	0	1	4.46	3.7	4.72
181	1	1	1	1	1	1	0	1	2.72	2.42	4.36
182	1	1	1	1	1	1	1	1	3.39	2.81	7
183	1	1	1	1	1	1	1	1	4.45	3.87	10.77
184	1	1	1	1	0	1	0	1	2.81	1.7	3
185	1	1	1	1	1	1	0	1	4.88	4.3	7.64
186	1	1	1	1	1	1	1	1	3.57	3	5.29
187	1	1	1	1	1	1	1	1	2.92	2.41	5.16
188	—	—	—	—	—	—	—	—	—	—	—
189	1	1	1	1	1	1	1	1	3.15	2.63	5.83
190	1	1	1	1	0	1	1	1	3.91	3.4	7.12
191	1	1	1	1	1	1	1	1	3.11	2.89	5.41
192	1	1	1	1	1	1	1	1	3.12	3.1	5.94
193	1	1	1	1	0	1	0	1	2.44	2.31	4.65
194	1	1	1	1	1	1	0	1	4.67	3.94	9.69
195	1	1	1	1	1	1	0	1	5.01	4.18	8.94
196	1	1	1	1	0	1	0	1	1.89	1.79	3.32
197	1	1	1	1	1	1	1	1	2.55	2.27	4.72
198	1	1	1	1	1	1	1	1	3.86	3.1	8.81
199	1	1	1	1	1	0	1	1	3.84	2.99	7.97
200	—	—	—	—	—	—	—	—	—	—	—
201	1	1	1	1	1	1	1	1	5.08	4.41	11.45
202	0	1	1	1	0	0	0	1	2.91	2.63	3.52
203	—	—	—	—	—	—	—	—	—	—	—
204	—	—	—	—	—	—	—	—	—	—	—
205	1	1	1	1	1	1	0	1	3.19	2.56	3.18
206	1	1	1	1	1	1	0	1	1.93	1.75	3.45
207	1	1	1	1	1	0	1	1	2.44	1.97	4.17
208	0	1	1	1	1	1	1	1	6.21	4.46	7.54
209	0	1	1	1	1	1	1	1	3.26	2.85	5.92
210	1	1	1	1	0	1	0	1	3.4	2.7	4.67
211	1	1	1	1	1	1	1	1	5.05	4.08	6.3
212	1	1	1	1	0	0	0	0	4.66	3.86	12.6
213	0	1	1	1	1	0	1	1	2.04	1.99	4.32
214	1	1	1	1	1	1	1	1	3.44	3.04	7.16
215	1	1	1	1	0	0	1	1	1.93	1.64	2.99
216	—	—	—	—	—	—	—	—	—	—	—
217	—	—	—	—	—	—	—	—	—	—	—
218	1	1	1	1	1	1	1	1	2	1.76	4.5
219	1	1	1	1	0	1	0	1	1.73	1.45	3.06
220	1	1	1	1	0	0	0	1	5.61	4.42	9.06
221	1	1	1	1	1	1	1	1	2.55	2.51	5.94
222	1	1	1	1	0	1	0	1	5.23	3.96	11.78
223	1	1	1	1	1	1	0	1	4.5	4.47	8.24
224	0	1	1	1	0	0	0	1	5.09	4.4	6.6
225	—	—	—	—	—	—	—	—	—	—	—
226	1	1	1	1	0	1	0	1	2.84	2.42	5.16
227	1	1	1	1	1	1	1	1	4.09	2.94	6.43
228	1	1	1	1	0	1	1	1	4.71	3.79	7.2
229	1	1	1	1	0	1	0	1	2.05	1.74	3.67
230	1	1	1	1	0	1	1	1	2.56	2.18	5.41
231	1	1	1	1	1	1	1	1	3.15	3.06	5.6
232	1	1	1	1	0	0	0	1	2.46	2.18	4.48
233	1	1	1	1	1	1	1	1	3.63	2.94	5.65
234	1	1	1	1	1	1	0	1	5.27	3.83	7.12
235	1	1	1	1	1	1	1	1	2.92	2.44	4.75
236	1	1	1	1	1	1	1	1	2.96	2.87	6.67
237	1	1	1	1	0	1	0	1	5.81	4.71	7.12
238	1	1	1	1	1	1	1	1	2.37	2.14	2.79
239	0	1	1	1	0	0	0	1	2.77	2.6	6.49
240	1	1	1	1	0	1	1	1	2.24	1.46	2.57
241	1	1	1	1	1	1	1	1	3.09	2.37	2.79
242	1	1	1	1	0	0	1	1	2.99	2.43	5.34
243	1	1	1	1	0	0	0	1	3.82	3	5.36
244	1	1	1	1	1	1	1	1	5.41	5.07	10
245	0	1	1	1	0	1	1	1	3.09	3.05	9.13
246	1	1	1	1	0	0	0	1	4.3	3.87	10.16
247	1	1	1	1	0	1	1	1	3.18	2.55	6.27
248	1	1	1	1	1	1	1	1	2.82	2.28	4.23
249	0	1	1	1	0	1	1	1	3.72	3.24	9.06
250	1	1	1	1	1	1	1	1	4.18	3.04	7.68
251	1	1	1	1	1	1	0	1	5.7	5.33	10.86
252	1	1	1	1	0	0	0	1	4.85	3.4	9.26
253	1	1	1	1	1	1	1	1	—	—	—
254	1	1	1	1	1	1	1	1	4.51	3.31	6.63
255	—	—	—	—	—	—	—	—	—	—	—
256	1	1	1	1	0	0	1	1	2.85	2.71	5.97
257	1	1	1	1	1	1	1	1	2.91	2.49	5.86
258	1	1	1	1	0	0	0	1	2.32	1.86	2.47
259	1	1	1	1	1	0	1	1	3.34	2.8	6.06
260	1	1	1	1	0	1	0	1	2.68	2.63	7.28
261	0	1	1	1	0	1	1	1	3.18	2.87	9.33
262	1	1	1	1	1	1	1	1	3.54	3.07	8.46
263	1	1	1	1	0	0	0	1	1.99	1.49	1.95
264	1	1	1	1	1	1	1	1	3.11	2.48	5.74
265	1	1	1	1	1	1	1	1	3.62	1.99	2.07
266	1	1	1	1	1	0	0	1	3.11	3.57	5.75
267	1	1	1	1	0	1	0	1	3.57	3.11	5.65
268	0	1	1	1	1	1	1	1	3.31	2.9	6.3
269	1	1	1	1	1	1	1	1	2.6	2.31	3.53
270	—	—	—	—	—	—	—	—	—	—	—
271	—	—	—	—	—	—	—	—	—	—	—
272	1	1	1	1	0	1	1	1	3.5	2.96	7.83
273	1	1	1	1	1	1	0	1	2.44	2.37	4.32
274	1	1	1	1	1	1	1	1	3.4	3.07	5.94
275	—	—	—	—	—	—	—	—	—	—	—
276	1	1	1	1	1	1	0	1	4.08	3.68	6.92
277	1	1	1	1	1	0	0	1	3.02	2.89	5.45
278	1	1	1	1	1	0	1	1	5.12	4.21	11.67
279	—	—	—	—	—	—	—	—	3.05	2.52	4.86
280	1	1	1	1	1	1	1	1	3.2	2.49	5.78
281	1	1	1	1	1	0	0	0	5.15	4.02	8.4
282	1	1	1	1	0	1	0	1	3.4	2.58	4.63
283	1	1	1	1	1	1	0	1	2.04	1.71	5.25
284	—	—	—	—	—	—	—	—	—	—	—
285	1	1	1	1	1	1	0	1	4.27	3.3	7.97
286	1	1	1	1	1	1	1	1	3.14	3.02	7.42
287	1	1	1	1	1	1	1	1	5.17	4.79	8.57
288	1	1	1	1	1	1	1	1	4.61	3.92	9.13
289	1	1	1	1	1	0	1	1	2.89	2.6	5.83
290	—	—	—	—	—	—	—	—	3.33	2.75	4.34
291	1	1	1	1	0	0	0	1	2.87	2.84	5.6
292	1	1	1	1	0	1	1	1	—	—	—
293	1	1	1	1	0	1	0	1	—	—	—

**Table 8:** **Data collected from visitors during the Days of Science and Technology 2016** ID - id of the participant, Pressure[mm/Hg] - blood pressure of the participant as systolic, diastolic blood pressure), Puls[puls/min] - heart rate calculated by Omron M6 Comfort IT, HR[puls/min] - heart rate calculated by ReadMyHeart Handheld ECG, ST-S[mm] - ST segment, QRS-I[s] - QRS interval, Glucose[mmol/l] - concentration of sugar in blood, “—” - blank data (data were not filled in).

ID	Pressure[mm/Hg]	Puls[puls/min]	HR[puls/min]	ST-S[mm]	QRS-I[s]	Glucose[mmol/l]
1	120, 70	69	63	0.35	0.06	—
2	105, 86	69	80	0.5	0.1	5.5
3	—	—	—	—	—	—
4	—	—	—	—	—	—
5	—	—	—	—	—	—
6	—	—	—	—	—	—
7	137, 85	63	87	0.18	0.08	6.5
8	—	—	—	—	—	—
9	—	—	—	—	—	—
10	—	—	—	—	—	—
11	95, 75	78	77	-0.5	0.05	6.5
12	124, 69	60	69	-0.04	0.12	6
13	122, 67	74	84	0.93	0.08	6.1
14	132, 77	59	66	-1.73	0.05	5.5
15	118, 83	106	107	0.51	0.18	6
16	—	—	—	—	—	—
17	—	—	—	—	—	—
18	—	—	—	—	—	—
19	—	—	—	—	—	—
20	110, 74	73	71	-0.28	0.1	5.3
21	98, 70	72	78	0.33	0.12	4.3
22	122, 72	72	73	1.61	0.12	4.7
23	—	—	—	—	—	—
24	122, 74	70	—	—	—	5.5
25	—	—	—	—	—	—
26	—	—	—	—	—	—
27	—	—	—	—	—	—
28	—	—	—	—	—	—
29	—	—	—	—	—	—
30	—	—	—	—	—	—
31	—	—	—	—	—	—
32	—	—	—	—	—	—
33	130, 76	78	78	0.65	0.05	6.8
34	128, 63	60	55	0.04	0.05	4.7
35	121, 68	62	66	2.18	0.1	5.5
36	119, 79	94	96	-1.15	0.08	7.6
37	105, 70	65	71	0.06	0.07	5.2
38	134, 68	101	83	-1.17	0.05	4.6
39	125, 86	85	84	-3.18	0.05	—
40	137, 69	75	—	—	—	5.2
41	115, 66	40	71	-0.65	0.05	5.2
42	—	—	—	—	—	—
43	118, 75	111	119	5.39	0.2	6
44	—	—	—	—	—	—
45	—	—	—	—	—	—
46	—	—	—	—	—	—
47	104, 61	88	81	1.99	0.15	—
48	116, 97	132	106	0	0.12	—
49	—	—	—	—	—	—
50	—	—	—	—	—	—
51	—	—	—	—	—	—
52	122, 72	106	—	—	—	5
53	—	—	—	—	—	—
54	—	—	—	—	—	—
55	—	—	—	—	—	—
56	—	—	—	—	—	—
57	—	—	—	—	—	—
58	—	—	—	—	—	—
59	112, 76	67	64	-0.51	0.09	5.3
60	—	—	—	—	—	—
61	—	—	—	—	—	—
62	135, 89	67	65	-2.13	0.08	6
63	—	—	—	—	—	—
64	—	—	—	—	—	—
65	—	—	—	—	—	—
66	155, 77	60	64	1.47	0.1	5.7
67	—	—	—	—	—	—
68	—	—	—	—	—	—
69	132, 77	81	87	3.46	0.13	—
70	136, 87	96	109	1.67	0.1	—
71	142, 84	71	73	0.13	0.1	5.7
72	111, 71	91	90	-1.46	0.05	6.6
73	118, 70	85	97	0.18	0.05	—
74	102, 73	74	87	0.82	0.08	—
75	95, 69	91	83	0.18	0.1	—
76	134, 78	65	112	-0.54	0.01	6.2
77	—	—	—	—	—	—
78	155, 82	75	78	-1.57	0.12	4.8
79	105, 65	56	57	0.42	0.01	6
80	—	—	—	—	—	—
81	—	—	—	—	—	—
82	122, 76	106	—	—	—	—
83	113, 63	80	—	—	—	—
84	—	—	—	—	—	—
85	—	—	—	—	—	—
86	146, 82	91	98	0.45	0.15	—
87	114, 82	82	75	-0.22	0.08	4.6
88	160, 87	73	75	1.5	0.05	7.3
89	140, 82	67	67	-2.51	0.05	5.7
90	112, 84	82	79	1.06	0.1	—
91	139, 98	76	74	-4.28	0.1	5.7
92	—	—	—	—	—	—
93	106, 79	86	92	1.75	0.1	5
94	92, 78	92	72	4.02	0.19	5.1
95	102, 73	94	99	-1.14	0.05	6
96	141, 89	68	66	-3.58	0.09	6.2
97	—	—	—	—	—	—
98	121, 94	79	76	-2.31	0.08	4.8
99	123, 76	70	77	-0.96	0.05	5.8
100	140, 90	97	98	-0.32	0.05	—
101	126, 87	68	66	-0.8	0.05	5.5
102	137, 87	64	71	0.94	0.08	5
103	108, 68	89	97	-0.18	0.05	—
104	105, 60	74	76	-0.43	0.08	—
105	—	—	118	0.62	0.08	—
106	92, 70	85	85	-1.9	0.08	—
107	110, 69	88	93	-1.47	0.05	—
108	128, 71	81	78	1.08	0.09	4.9
109	105, 66	74	70	-1.5	0.08	5.1
110	121, 78	71	70	1.47	0.05	5.8
111	—	—	—	—	—	—
112	115, 101	99	92	-2.28	0.05	7.1
113	129, 88	79	72	-0.97	0.05	6.3
114	124, 82	73	80	0.42	0.08	4.4
115	91, 70	84	82	-0.07	0.09	—
116	—	—	—	—	—	—
117	124, 94	73	76	0.47	0.07	5.3
118	101, 84	54	89	1.92	0.09	—
119	102, 76	79	83	0.33	0.08	6.7
120	101, 68	81	78	-0.8	0.07	6.4
121	98, 82	92	—	—	—	—
122	97, 64	93	93	0.61	0.1	—
123	—	—	96	1.95	0.1	—
124	171, 98	70	73	-0.09	0.11	5.3
125	110, 91	88	87	-0.67	0.1	—
126	115, 75	74	72	0.24	0.07	—
127	129, 85	85	74	0.33	0.1	5.5
128	82, 66	103	98	0.99	0.08	—
129	107, 81	79	90	0.7	0.09	—
130	122, 85	104	98	-0.04	0.4	6.6
131	126, 73	65	65	0.99	0.1	4.7
132	99, 75	72	80	-1.12	0.05	4.8
133	143, 83	54	60	0.19	0.1	5.3
134	113, 85	63	—	—	—	5.4
135	126, 78	59	—	—	—	5.5
136	98, 65	77	64	0.97	0.08	6.1
137	—	—	97	0.7	0.07	—
138	120, 84	62	58	1.63	0.11	5.2
139	98, 73	78	82	0.59	0.09	5.8
140	—	—	—	—	—	—
141	140, 85	80	80	0.27	0.1	6
142	112, 74	74	68	0.04	0.08	5.5
143	138, 76	71	71	1.81	0.1	6.6
144	102, 70	70	67	-0.38	0.98	5.5
145	125, 76	79	72	-1.57	0.1	4.7
146	163, 105	70	69	-0.2	0.104	5.2
147	113, 78	69	61	0.48	0.112	5.3
148	112, 84	68	217	-0.37	0.084	4.5
149	110, 86	97	172	-1.02	0.1	5.3
150	151, 77	58	53	2.36	0.12	4.6
151	107, 70	63	112	0.17	0.104	4.7
152	122, 77	71	97	0.31	0.132	4.8
153	96, 62	61	70	0.61	0.108	—
154	160, 84	65	59	-0.1	0.116	5.1
155	129, 85	65	217	0.17	0.184	5.6
156	—	—	—	—	—	—
157	136, 71	49	50	0.37	0.12	4.7
158	126, 84	68	72	0.73	0.116	5.7
159	89, 67	70	71	0.26	0.116	—
160	—	—	—	—	—	—
161	106, 66	73	93	2	0.116	—
162	—	—	81	0.24	0.104	—
163	115, 74	77	77	0.14	0.12	—
164	—	—	—	—	—	—
165	119, 76	73	90	-0.9	0.076	5.6
166	124, 85	71	78	0.39	0.112	11.4
167	109, 76	83	79	1.91	0.172	5.3
168	121, 71	83	85	0.54	0.104	—
169	102, 79	81	87	2.8	0.172	5.3
170	126, 83	90	94	1.22	0.112	6.3
171	114, 74	87	94	1.22	0.122	4.8
172	128, 81	76	—	—	—	5.1
173	107, 74	85	81	0.88	0.108	—
174	120, 80	97	107	0.55	0.108	—
175	92, 75	94	—	—	—	—
176	—	—	—	—	—	—
177	—	—	—	—	—	—
178	106, 89	81	96	1.72	0.176	—
179	119, 77	77	78	0.75	0.108	5.8
180	145, 81	89	100	1.07	0.109	—
181	97, 62	87	98	1.53	0.164	—
182	107, 71	79	82	1.75	0.196	5.7
183	121, 86	65	64	0.03	0.104	5.7
184	—	—	—	—	—	—
185	146, 86	75	74	1.9	0.176	7.2
186	119, 92	89	61	2.07	0.172	5.6
187	127, 83	70	95	0.17	0.112	4.5
188	—	—	—	—	—	—
189	142, 83	84	83	-0.1	0.132	6.3
190	135, 92	93	111	1.46	0.192	4.7
191	107, 71	71	81	0.47	0.116	—
192	134, 85	102	104	0.85	0.112	5.7
193	129, 67	55	—	—	—	7.9
194	154, 89	56	—	—	—	5.2
195	116, 81	52	88	1.84	0.128	4.8
196	97, 62	49	100	2.9	0.168	—
197	163, 83	67	82	-0.47	0.108	6.5
198	118, 70	73	—	—	—	5.4
199	113, 84	54	76	0.12	0.112	5.2
200	—	—	—	—	—	—
201	127, 67	66	70	1.64	0.112	5.6
202	104, 64	99	—	—	—	4.7
203	174, 68	94	—	—	—	5.1
204	131, 76	76	—	—	—	4.8
205	143, 100	78	71	0.78	0.189	5.3
206	87, 69	77	74	0.62	0.112	—
207	128, 85	86	88	2.07	0.168	6.5
208	107, 77	76	123	0.4	0.188	—
209	139, 90	89	81	0.5	0.084	5.8
210	102, 69	66	80	1.12	0.2	—
211	125, 78	61	69	0.37	0.132	—
212	127, 82	80	—	—	—	—
213	110, 83	81	91	1.57	0.16	8.1
214	113, 85	78	75	-0.11	0.108	—
215	124, 74	100	98	-0.59	0.084	—
216	—	—	—	—	—	—
217	—	—	—	—	—	—
218	136, 83	76	76	-1.74	0.088	—
219	89, 71	96	85	0.28	0.152	—
220	125, 79	75	96	1.37	0.184	6.1
221	119, 79	77	102	0.92	0.172	—
222	129, 77	90	86	2.49	0.16	7.4
223	110, 73	55	90	0.01	0.12	4.7
224	105, 62	64	63	1.17	0.112	4.9
225	—	—	—	—	—	—
226	130, 85	87	83	0.23	0.092	4.8
227	110, 71	74	70	0.85	0.176	6.1
228	119, 78	79	—	—	—	5.6
229	82, 64	98	—	—	—	6.6
230	142, 78	94	79	-0.2	0.084	7.2
231	123, 96	92	96	0.93	0.148	4.8
232	109, 62	86	87	-0.12	0.084	7
233	80, 61	86	86	0.45	0.108	5.6
234	110, 67	69	78	0.97	0.116	—
235	74, 60	86	92	0.46	0.104	—
236	148, 91	83	81	—	—	9.6
237	131, 76	82	87	0.78	0.104	—
238	130, 76	70	72	-0.4	0.084	8.3
239	157, 69	88	98	-1.64	0.104	7
240	116, 100	115	110	0.36	0.108	—
241	108, 89	105	104	1.46	0.108	—
242	141, 82	66	95	0.9	0.176	8.7
243	129, 81	85	86	0.63	0.108	5.2
244	158, 80	82	85	2.63	0.156	6.5
245	108, 66	83	97	-0.01	0.116	5.3
246	136, 78	81	82	0.19	0.108	6.1
247	111, 73	60	66	-0.43	0.104	7.8
248	114, 76	94	103	-0.5	0.084	8.2
249	113, 77	52	52	2.11	0.184	8.2
250	124, 78	85	75	1.47	0.152	6.4
251	122, 67	83	—	—	—	6.8
252	126, 72	80	81	3.06	0.164	6
253	102, 74	93	135	1.79	0.196	5.6
254	117, 80	95	93	1.12	0.104	6
255	—	—	—	—	—	—
256	134, 79	80	72	-0.84	0.16	7.8
257	100, 81	127	122	-0.08	0.112	5
258	—	—	93	2.33	0.176	—
259	106, 75	97	92	0.69	0.184	8.1
260	147, 87	78	76	-0.95	0.088	5.1
261	102, 73	109	102	1.06	0.108	5.5
262	143, 88	82	78	3.03	0.112	—
263	113, 61	90	92	0.93	0.112	—
264	98, 65	87	94	1.5	0.1	—
265	129, 91	85	81	-0.57	0.084	5.2
266	127, 72	69	—	—	—	—
267	110, 73	79	73	0.51	0.152	—
268	126, 90	68	72	-0.39	0.112	5.1
269	104, 89	107	119	1.09	0.192	5.2
270	—	—	—	—	—	—
271	—	—	—	—	—	—
272	142, 95	97	72	0.37	0.112	6.2
273	93, 62	68	74	0.76	0.108	—
274	121, 88	75	70	0.64	0.104	5.3
275	—	—	—	—	—	—
276	180, 97	93	73	0.36	0.108	13.7
277	108, 68	88	78	0.61	0.124	6.3
278	152, 89	97	100	0.72	0.108	4.8
279	109, 78	82	76	0.02	0.088	5.6
280	132, 89	105	97	0.73	0.16	5.8
281	122, 71	57	56	1.89	0.124	5.4
282	148, 89	59	59	0.96	0.188	6.1
283	129, 70	83	70	-1.27	0.084	6.1
284	—	—	—	—	—	—
285	109, 80	81	71	0.85	0.104	6.1
286	130, 86	114	84	0.31	0.096	4.6
287	130, 83	82	79	0.4	0.12	5.3
288	114, 66	63	70	0.87	0.112	5.6
289	107, 77	81	77	0.51	0.124	5.6
290	90, 55	63	60	-0.78	0.088	6.1
291	118, 71	80	79	—	—	—
292	—	—	—	—	—	—
293	—	—	—	—	—	—

**Table 9:** **Data collected from visitors during the Days of Science and Technology 2016** ID - index of the participant, Height[cm] - height of the participant, Weight[kg] - weight of the participant, BMI - body mass index, Muscle-mass[%] - percentage of muscles in the body, Water[%] - percentage of water in the body, Fat[%] - percentage of fat in the body, Flexibility[cm] - how much the participant was able to bend (negative - could not even touch their feet, positive - bent even further), “—” - blank data (data were not filled in).

ID	Height[cm]	Weight[kg]	BMI	Muscle-Mass[%]	Water[%]	Fat[%]	Flexibility[cm]
1	170	63.5	22	47.7	63.4	13.5	3
2	—	—	—	—	—	—	—
3	—	—	—	—	—	—	—
4	—	—	—	—	—	—	—
5	—	—	—	—	—	—	—
6	—	—	—	—	—	—	—
7	156	66.2	27.2	25	51.8	36.3	1
8	—	—	—	—	—	—	—
9	—	—	—	—	—	—	—
10	—	—	—	—	—	—	—
11	167	65.4	23.5	33.7	55.7	22.2	13
12	185	72.2	21.1	53.6	67.1	7.1	7
13	179	69.9	21.8	53.4	66.4	8.1	13
14	172	67.1	22.7	52.3	65.4	9.5	13
15	170	68.8	23.8	36.8	51.1	24.7	13
16	—	—	—	—	—	—	—
17	—	—	—	—	—	—	—
18	—	—	—	—	—	—	—
19	—	—	—	—	—	—	—
20	143	30.6	15	57.9	70.7	5	5
21	162	44	16.8	57.6	71.7	5	-7
22	178	86.8	27.4	46.4	56.8	20.2	4
23	—	—	—	—	—	—	—
24	173	68.2	22.8	29.6	54.3	26.2	13
25	—	—	—	—	—	—	—
26	—	—	—	—	—	—	—
27	—	—	—	—	—	—	—
28	155	55.6	23.1	37.5	51.7	23.7	-7
29	163	45.3	17	42	58.7	14.3	13
30	156	48.3	19.8	40.1	55.6	18.3	13
31	—	—	—	—	—	—	—
32	—	—	—	—	—	—	—
33	165	71.7	26.3	31.2	49.1	30.2	-3
34	177	65.6	20.9	38	54.5	20.7	10
35	193	84.6	22.7	50	62.4	13.5	13
36	174	65.9	21.8	37.7	53.6	21.7	13
37	160	54.5	21.3	38	54	21.2	13
38	168	58.2	20.6	38.1	54.8	20.4	13
39	172	71.4	24.1	45.3	61.2	16.9	11
40	180	72.5	22.4	50.5	62.5	13.1	12
41	175	57.4	18.7	32.2	59.1	20	13
42	—	—	—	—	—	—	—
43	182	77.3	22	50.1	63.2	12.7	0
44	—	—	—	—	—	—	—
45	—	—	—	—	—	—	—
46	—	—	—	—	—	—	—
47	168	62.5	22.1	50.8	62.9	12.6	11
48	144	73.8	19.6	52.6	65.6	9.1	10
49	—	—	—	—	—	—	—
50	—	—	—	—	—	—	—
51	—	—	—	—	—	—	—
52	—	—	—	—	—	—	—
53	—	—	—	—	—	—	—
54	—	—	—	—	—	—	—
55	—	—	—	—	—	—	—
56	—	—	—	—	—	—	—
57	—	—	—	—	—	—	—
58	—	—	—	—	—	—	—
59	166	63.6	23.1	34	52.8	24.8	13
60	—	—	—	—	—	—	—
61	—	—	—	—	—	—	—
62	168	89	31.5	25	46	41.6	2
63	—	—	—	—	—	—	—
64	—	—	—	—	—	—	—
65	—	—	—	—	—	—	—
66	186	104	30.1	30	56.7	28.2	-17
67	—	—	—	—	—	—	—
68	—	—	—	—	—	—	—
69	185	77.9	22.8	49.9	62.3	13.6	7
70	184	78.7	23.2	49.3	61.5	14.6	-10
71	168.5	98.6	34.5	25	43	44.6	0
72	172	74.9	25.3	28.6	51.2	29.8	10
73	175.5	56.8	18.3	40.6	57.4	16.4	13
74	176	64.6	20.9	39	54.3	20.3	6
75	164	59.2	22	37.4	53.5	22	6
76	163	55.3	20.8	28.7	57.1	24.1	13
77	—	—	—	—	—	—	—
78	161	61.6	23.8	25	54.8	30.1	-1
79	165	77.4	28.4	25.5	59.9	27.3	-14
80	—	—	—	—	—	—	—
81	—	—	—	—	—	—	—
82	184	89.3	26.4	47.9	57.6	19.1	-9
83	171	63.1	21.6	38.4	53.7	21.2	12
84	—	—	—	—	—	—	—
85	—	—	—	—	—	—	—
86	178	68.9	21.7	36.9	53	22	15
87	165	66.1	24.3	26.6	53.2	29.1	-3
88	162	95.7	36.5	25	43	50.4	-15
89	187	80.5	23.8	31.8	64.7	19.8	6
90	153	53.6	22.9	51.7	61.3	13.7	6
91	161	96.9	37.4	25	43	47.5	13
92	—	—	—	—	—	—	—
93	148	37.4	17.1	56.9	68.1	5	-4
94	152	41.6	18	56.3	67.2	6	-12
95	170	55.5	19.2	38.6	56.5	18.5	13
96	183	9836	29.4	27.4	57.9	28.2	-18
97	—	—	—	—	—	—	—
98	—	—	—	—	—	—	-2
99	170	75.1	26	37	60.7	21.2	9
100	175	57.3	18.7	52.7	70.3	5	13
101	176	62.9	20.3	28.1	58.1	23.6	-19
102	182	82.2	24.8	32.5	63.2	20.9	3
103	176	58.7	19	40.2	56.7	17.3	0
104	156	51.1	21	38.8	54.4	20.3	11
105	147	34.1	15.8	42.8	60	12.5	-3
106	150	43.3	19.2	40	56.3	17.7	13
107	168	59	20.9	38.5	54.4	20.5	4
108	186	80.5	23.3	51.8	64.7	10.4	13
109	162	56.8	21.6	37.1	54	21.8	13
110	167	68	24.4	25	53.8	30.2	13
111	—	—	—	—	—	—	—
112	162	52.4	20	35	56.4	20.8	-5
113	186	89	25.7	43.7	59.5	19.2	-11
114	176	63.6	20.5	32.2	56.6	22.4	-5
115	141	32.8	16.5	42.8	59.1	13.4	13
116	—	—	—	—	—	—	—
117	166	66	24	28.3	53	28.2	13
118	117	23.3	17	42.6	58.7	13.8	-15
119	167	95.1	34.1	25	43	43.1	8
120	169	60.2	21.1	31.3	56.1	23.4	13
121	126	26.8	16.9	57.1	68.4	5	0
122	152	38.8	16.8	57.1	68.4	5	—
123	122	23.6	15.9	57.7	69.3	5	—
124	185	106.2	31	39.5	53.4	26.9	0
125	156	73.1	30	25	46	36.9	11
126	154	54	22.8	37.3	52.4	23.1	13
127	180	93.7	28.9	34.7	57.3	25.5	2
128	146	37.9	17.8	41.2	57.8	15.6	8
129	169	80.7	28.3	32.9	46.2	31.8	8
130	165	85.2	31.3	30.9	43	36	11
131	177	75.9	24.2	46.5	61	16.5	6
132	167	50.2	18	38.1	58.2	17.2	0
133	172	81.1	27.4	38.4	58.5	22.5	-6
134	172	69.7	23.6	29.7	53.1	27.3	0
135	—	—	—	—	—	—	0
136	160	60.7	23.7	30.7	52.7	27	3
137	115	20.2	15.3	39.6	61.6	13	6
138	193	95.4	25.6	44.3	59.6	18.8	0
139	171	61.7	21.1	35.9	55.1	21.5	7
140	—	—	—	—	—	—	—
141	191	95.7	26.2	44.9	58.8	19.3	10
142	180	67.4	20.8	39.4	57.9	16.7	11
143	190	88	24.4	48.6	63.8	12.7	13
144	167	63.9	22.9	34.2	53.2	24.3	12
145	186	81.4	23.5	45.3	62.3	15.8	-9
146	180	117.2	36.2	25	50.7	38.7	3
147	185	68.9	20.1	51.5	65.1	10.1	-5
148	165	52.4	19.2	38.5	56.8	18.3	8
149	163	55.5	20.9	35.9	55.9	21.5	12
150	180	84.4	26	44.3	59.1	19.3	0
151	178	78.9	24.9	28.2	52	29.2	2
152	174	68.6	22.7	27	54.9	27.3	12
153	160	42.2	16.5	56	68.7	5	-4
154	172	80.5	27.2	26.5	61.3	25.6	4
155	186	84.9	23.8	32.9	64.5	19.5	5
156	—	—	—	—	—	—	—
157	160	75.8	29.6	25	58.6	29.3	1
158	175	90.9	29.7	36.6	55.8	26	-14
159	142	33.7	16.7	56.9	68.2	5	5
160	140	35.7	18.2	41.3	57.1	16.1	-1
161	166	56.5	20.5	39	54.7	19.9	6
162	137	28.8	15.3	43.5	60.2	11.8	-12
163	185	67.4	19.7	52.2	65.6	9.3	-23
164	—	—	—	—	—	—	—
165	164	79.9	29.7	29.4	45.3	34.8	8
166	182	106	32	30.7	54	30.5	-21
167	168	78.1	27.7	26.2	48.7	33.6	10
168	170	66.5	23	50.4	61.8	13.9	-4
169	—	—	—	—	—	—	4
170	163	90.1	33.9	25.5	43	41.3	-4
171	164	55.7	20.7	32.8	56.2	22.3	9
172	177	76.9	24.5	43.6	61.1	17.7	-12
173	165	68.7	25.2	35.8	49.4	27	-5
174	145	40.8	19.4	40.8	55.9	17.6	13
175	140	25.1	12.8	45.3	63.2	7.8	-13
176	—	—	—	—	—	—	—
177	—	—	—	—	—	—	—
178	155	41.7	17.4	55.5	67.9	5.6	-19
179	169	88.9	31.1	25	44.6	38.1	0
180	190	77.1	21.4	52.1	63.7	11.3	13
181	153	41.5	17.7	41	58	15	8
182	161	59.4	22.9	29.5	54.1	26.5	-2
183	172	69.2	23.4	40.8	63.1	17.1	-10
184	125	26.8	17.2	56.9	68.1	5	0
185	177	104.9	33.5	29.1	52.4	32.8	12
186	176	70.4	22.7	30	54	26.3	12
187	163	52.6	19.8	30.2	58.2	22.2	-4
188	—	—	—	—	—	—	—
189	170	106.2	36.7	25	43	47.7	12
190	181	84.4	25.8	44.7	59.1	19.1	-10
191	162	54.2	20.7	38.7	54.8	20	13
192	165	76.9	28.2	31.5	46.8	32	13
193	172	77.1	26.1	26.7	62.7	24.1	-12
194	197	98.6	25.4	35.6	61.6	21	3
195	167	79.7	28.6	36.7	57.3	24.5	4
196	134	27	15	58.5	70.4	5	7
197	167	82.9	29.7	25	48.2	34.2	12
198	173	64.9	21.7	33.4	58.3	19.9	13
199	178	71.7	22.6	40.4	64.4	16	10
200	—	—	—	—	—	—	—
201	182	84.9	25.6	43.9	59.8	18.8	0
202	167	65.2	23.4	36.8	51.6	24.3	5
203	—	—	—	—	—	—	—
204	—	—	—	—	—	—	—
205	164	54.9	20.4	32.6	56.8	22	13
206	139	29	15	58.5	70.5	5	6
207	168	95.8	33.9	25	43.5	45.5	10
208	196	88.5	23	47.4	62.3	14.7	8
209	171	63.3	21.6	32.7	55.1	23.5	13
210	168	51.3	18.2	37.5	58.2	17.6	4
211	178	75.1	23.7	43.6	62.4	16.5	13
212	179	76.7	23.9	36	63.5	19	8
213	165	72.6	26.7	26.5	50.1	32.1	9
214	168	96.7	34.3	25.3	43	41.9	11
215	163	85.8	32.3	25	45.3	43	13
216	—	—	—	—	—	—	—
217	—	—	—	—	—	—	—
218	159	61.8	24.4	25	54.9	31.9	13
219	130	27.4	16.2	57.6	69.1	5	8
220	180	84.6	26.1	38.6	60	21.1	0
221	170	56.8	19.7	36.4	56.8	19.6	0
222	184	119.9	35.4	29.8	49.7	35	-23
223	182	67.5	20.4	47.9	65.4	11.6	0
224	175	72	23.5	41.9	62.9	16.8	13
225	—	—	—	—	—	—	—
226	164	63.8	23.7	27.8	53.3	28.4	8
227	187	96.7	27.7	34.1	58.9	24.3	0
228	175	61.3	20	32.5	57.1	21.8	0
229	145	38.6	18.4	41.5	57	16.2	0
230	156	72	29.6	25	47.5	38.4	4
231	176	68.1	22	35	53.9	23.2	0
232	158	63.9	25.6	25	52.2	32.4	12
233	173	52.1	17.4	33.1	60.5	18.2	-1
234	185	63.2	18.5	53.6	67.1	7.3	-17
235	164	50.8	18.9	40.4	56.6	17.2	13
236	178	99.9	31.5	31	54.5	29.9	13
237	178	68.3	21.6	51.3	63.4	11.8	0
238	165	66.6	24.5	25	54.1	31.3	-12
239	158	70.1	28.1	25	49.6	36	13
240	163	59.9	22.5	37.2	52.7	23	10
241	161	90.4	34.9	28.9	43	41.3	2
242	178	89.5	28.2	24.4	60	26.8	-24
243	170	63.5	22	35.5	53.8	22.9	13
244	175	85.2	27.8	43.6	56.9	21.6	2
245	168	62.9	22.3	32.8	54.2	24.2	13
246	178	80.4	25.4	36.4	61.6	20.6	13
247	160	73.1	28.6	25	48.5	36.8	8
248	167	67.7	24.3	25	53.4	30.2	9
249	175	86.9	28.4	38.3	57.1	24	0
250	166	60.7	22	35.3	53.9	22.9	13
251	193	98	26.3	44.4	58.7	19.6	3
252	175	84.4	27.6	43.7	57	21.6	-3
253	162	50.4	19.2	30.8	58.7	21.2	-14
254	178	85	26.8	39.2	59.1	21.7	0
255	—	—	—	—	—	—	—
256	163	139.1	52.4	25	43	60	-5
257	156	85.4	35.4	25	43	43.1	-4
258	140	27.7	14.1	59.3	71.5	5	-10
259	160	62.9	24.6	40.1	61.6	18.9	6
260	167	89.3	32	25	44.1	40.1	10
261	169	84.7	29.7	25.6	46.3	36.2	—
262	188	97.2	27.5	38.4	58.4	22.7	10
263	150	50.8	22.6	38.6	52.2	22.5	-4
264	152	54.9	23.8	37.6	51	24.2	4
265	162	56.9	21.7	30.4	55.6	24.5	13
266	172	63.3	21.4	51.4	63.8	11.5	0
267	173	85.3	28.5	25	47.9	35	13
268	165	61.1	22.4	31	54.4	25.1	13
269	169	60.7	21.3	37	54	21.5	-26
270	—	—	—	—	—	—	—
271	—	—	—	—	—	—	—
272	168	80.9	28.7	26	47.5	34	13
273	170	54.3	18.8	40.3	56.8	17.1	0
274	164	60.9	22.6	33.8	53.4	24.5	13
275	—	—	—	—	—	—	—
276	180	93.5	28.9	33.2	57.6	25.9	-2
277	169	59.4	20.8	38.4	54.7	20.3	13
278	186	91.8	26.6	35.4	60.2	23.4	-10
279	170	63.7	22	31	55.2	24.5	—
280	173	70.5	23.6	28	53	27.3	11
281	182	80.4	24.3	43.2	61.7	17.4	-20
282	175	77.1	25.2	26.6	63.9	23	-34
283	156	68.2	28	25	51	37.8	8
284	—	—	—	—	—	—	—
285	183	83.8	25	41	60.8	19.1	-1
286	183	55.5	16.6	38.5	59.8	15.3	-5
287	180	74.1	22.9	45.7	62.9	15	0
288	183	82.2	24.5	46.7	60.7	16.7	-10
289	162	49.9	19	33	58.3	19.9	13
290	162	68	25.9	29.6	50.4	29.9	—
291	—	—	—	—	—	—	-9
292	—	—	—	—	—	—	0
293	—	—	—	—	—	—	6

**Table 10:** **Questionnaire data collected from visitors during the Days of Science and Technology 2016** ID - id of the participant, Q1-Q13 - Questions provided in Section 2.3, Q1-Q7 - 1 means “yes”, 0 means “no”, Q8 - 2 means “occasionally”, 1 means “yes”, 0 means “no”, Q9 - 0 means “up to 10 cigarettes per day”, 1 means “up to 10 cigarettes per week”, 2 means “up to 20 cigarettes per day”, 3 means “up to 10 cigarettes per month”, 4 means “20 or more cigarettes per day”, Q10 - 0 means “every day”, 1 means “once per week”, 2 means “multiple times per week”, 3 means “Occasionally”, 4 means “i don't consume any alcohol”, Q11-Q13 - 1 means “yes”, 0 means “no”, “—” - blank data (data were not filled in).

ID	Q1	Q2	Q3	Q4	Q5	Q6	Q7	Q8	Q9	Q10	Q11	Q12	Q13
1	1	—	—	1	0	1	0	0	—	4	1	0	0
2	1	—	—	1	0	1	0	0	—	2	1	1	0
3	1	—	—	1	0	1	0	1	—	2	1	1	0
4	1	—	—	1	0	1	0	0	—	4	1	0	1
5	1	—	—	1	1	0	0	0	—	4	1	0	1
6	1	—	—	1	1	1	0	1	0	3	1	0	1
7	0	1	0	1	0	1	1	0	—	4	0	0	1
8	1	—	—	1	0	1	0	1	0	4	1	0	0
9	1	—	—	1	1	1	1	0	—	4	1	0	1
10	1	—	—	1	1	0	1	2	3	4	0	1	0
11	0	1	1	0	1	1	1	0	—	4	1	1	0
12	1	—	—	1	1	1	1	0	—	3	1	0	1
13	1	—	—	0	1	1	0	0	—	4	1	0	1
14	1	—	—	1	1	1	1	0	—	3	1	0	1
16	1	—	—	1	1	1	0	0	—	4	1	0	0
15	1	—	—	1	0	1	0	0	—	4	1	0	1
17	1	—	—	0	0	1	0	0	—	4	1	0	1
18	1	—	—	1	1	1	1	0	—	4	1	0	1
19	0	1	1	1	1	0	1	0	—	4	1	0	1
20	1	—	—	1	1	1	0	0	—	4	1	1	0
21	0	0	—	1	1	1	0	0	—	4	1	0	1
22	1	—	—	1	1	1	1	0	—	3	1	0	1
23	0	1	1	1	1	0	0	0	—	4	1	0	1
24	0	1	1	1	0	1	1	0	—	3	1	1	1
25	1	—	—	1	1	1	1	0	—	4	1	0	1
26	1	—	—	1	1	1	1	0	—	4	1	0	1
27	1	—	—	1	1	1	1	0	—	4	1	0	1
28	1	—	—	1	0	0	0	0	—	4	1	0	1
29	1	—	—	0	0	0	0	0	—	4	1	0	1
30	1	—	—	1	0	0	0	0	—	4	1	0	1
31	1	—	—	1	0	0	0	0	—	4	0	0	1
32	1	—	—	1	1	1	0	0	—	4	1	0	0
33	0	1	1	1	1	1	0	0	—	3	1	1	1
34	1	—	—	1	1	1	1	0	—	4	1	1	1
35	1	—	—	1	0	1	1	0	—	3	1	0	0
36	1	—	—	1	0	1	0	0	—	4	1	0	1
37	1	—	—	1	1	1	0	2	3	3	1	0	1
38	0	1	1	0	0	1	0	0	—	4	1	0	1
39	1	—	—	1	1	0	0	0	—	3	1	1	0
40	1	—	—	0	1	0	1	0	—	3	1	0	1
41	1	—	—	1	0	1	1	0	—	2	1	1	1
42	1	—	—	1	0	0	1	0	—	4	1	0	1
43	1	—	—	0	1	1	0	2	3	3	1	0	1
44	1	—	—	1	0	1	0	0	—	4	1	0	0
45	1	—	—	1	1	1	0	0	—	4	1	0	0
46	1	—	—	1	1	1	0	0	—	1	1	1	1
47	1	—	—	1	1	1	1	0	—	3	1	1	1
48	1	—	—	1	1	1	1	0	—	3	1	0	1
49	0	1	1	1	1	1	1	2	0	3	1	1	1
52	0	0	—	1	1	1	0	1	0	4	0	0	0
53	1	—	—	1	1	1	1	0	—	4	1	1	0
54	1	—	—	1	1	1	0	0	—	4	1	0	0
55	0	1	1	1	1	1	0	0	—	4	1	0	1
56	0	1	1	1	1	1	0	0	—	4	1	0	1
57	0	1	1	1	1	1	1	0	—	3	1	0	1
58	0	1	1	1	0	1	0	0	—	4	1	0	0
59	1	—	—	1	1	1	1	1	2	3	1	0	0
60	0	1	1	1	0	0	0	0	—	3	1	1	0
61	1	—	—	1	0	1	1	0	—	3	1	1	1
62	0	0	—	1	1	1	0	0	—	4	1	0	1
63	1	—	—	1	1	1	1	0	—	4	1	0	1
64	1	—	—	1	1	1	1	0	—	4	1	0	1
65	1	—	—	1	1	1	0	0	—	4	1	0	0
66	0	1	0	1	1	1	0	0	—	3	1	1	0
67	0	1	0	1	0	1	1	0	—	4	1	0	1
68	1	—	—	1	1	1	0	0	—	4	1	0	1
69	1	—	—	0	1	0	0	0	—	2	0	0	1
70	0	1	1	0	0	1	0	0	—	3	1	0	1
71	1	—	—	0	1	0	0	0	—	3	0	1	1
72	0	1	0	1	0	0	1	0	—	3	1	1	1
73	1	—	—	1	0	1	0	0	—	3	1	0	1
75	1	—	—	1	1	1	0	0	—	4	0	0	1
74	1	—	—	0	1	0	0	0	—	4	1	0	0
76	1	—	—	0	1	1	0	0	—	2	1	1	0
77	1	—	—	1	1	1	1	0	—	4	1	0	0
78	1	—	—	1	0	1	0	0	—	0	1	1	1
79	0	0	—	1	0	1	0	0	—	0	1	1	1
80	1	—	—	1	1	1	1	0	—	4	1	0	1
81	1	—	—	1	1	1	0	0	—	4	1	0	1
82	1	—	—	0	1	1	1	0	—	3	1	1	1
83	1	—	—	1	0	1	1	0	—	4	1	1	1
84	0	1	1	1	1	1	0	0	—	4	1	0	1
85	1	—	—	1	1	1	0	0	—	4	1	0	1
86	0	0	—	0	1	1	1	1	0	4	1	1	1
87	0	1	1	1	1	0	1	0	—	4	1	1	1
88	1	—	—	1	0	0	1	0	—	3	1	0	1
89	1	—	—	0	0	0	0	0	—	0	0	1	1
90	0	0	—	1	1	0	0	0	—	4	1	0	1
91	0	1	1	1	0	1	0	0	—	3	1	1	1
92	0	1	1	1	1	1	1	0	—	0	1	0	1
93	1	—	—	1	1	1	0	0	—	4	1	0	1
94	1	—	—	1	1	1	0	0	—	4	0	0	1
95	0	1	1	0	0	1	0	0	—	1	1	0	1
96	0	1	1	1	1	1	0	0	—	2	1	1	1
97	0	1	1	1	1	1	1	0	—	3	1	0	1
98	0	1	0	0	0	0	1	0	—	1	1	1	0
99	0	1	1	1	0	0	0	0	—	4	1	1	1
100	1	—	—	1	1	1	0	0	—	4	0	0	1
101	0	1	0	0	0	1	0	0	—	3	0	1	1
102	0	1	1	1	0	0	0	0	—	3	0	1	0
103	0	1	1	1	0	1	0	0	—	4	1	0	1
104	1	—	—	1	1	1	0	0	—	4	1	0	1
105	0	1	1	1	0	1	0	0	—	4	1	0	1
106	0	1	1	1	0	1	0	0	—	4	1	0	1
107	0	0	—	1	0	1	1	0	—	4	1	0	1
108	1	—	—	1	1	1	1	0	—	3	1	1	1
109	0	0	—	1	1	1	0	0	—	3	0	1	0
110	0	1	1	0	0	1	1	0	—	3	0	0	0
112	0	1	1	1	0	1	0	0	—	1	0	1	1
113	0	1	1	1	1	0	0	1	4	4	1	1	1
114	0	1	1	1	0	1	0	0	—	4	1	1	0
115	1	—	—	1	1	1	0	0	—	4	1	0	1
116	0	1	1	1	1	1	0	1	0	4	1	0	1
117	0	1	0	0	0	1	0	0	—	4	0	1	0
118	0	1	1	1	1	1	1	0	—	4	1	0	1
119	0	1	1	1	1	1	1	1	0	3	1	1	1
120	0	0	—	1	0	1	0	0	—	3	1	1	1
121	1	—	—	1	0	1	0	0	—	4	1	0	1
122	1	—	—	1	0	1	0	0	—	4	1	0	1
123	1	—	—	1	0	1	0	0	—	4	1	0	1
124	0	1	1	1	1	1	0	0	—	2	0	0	1
125	0	1	0	1	0	1	0	0	—	4	1	0	1
126	0	0	—	1	0	0	0	0	—	1	1	1	1
127	0	0	—	1	1	1	0	0	—	3	0	1	0
128	1	—	—	0	1	0	0	0	—	4	1	0	1
129	0	1	1	1	1	0	0	0	—	4	1	1	1
130	0	1	1	0	0	1	1	2	0	3	1	0	0
131	1	—	—	1	1	1	1	2	3	3	1	1	1
132	0	1	1	0	0	0	1	2	3	3	1	1	1
133	1	—	—	1	1	1	0	0	—	2	1	0	1
134	0	1	0	1	0	1	0	0	—	3	0	1	0
136	1	—	—	1	0	1	0	0	—	1	1	1	1
137	1	—	—	1	0	1	0	0	—	4	1	0	1
138	0	0	—	1	1	0	0	0	—	2	1	1	1
139	0	1	1	0	0	1	0	0	—	1	1	1	1
140	0	1	0	0	0	0	1	0	—	3	1	1	1
141	1	—	—	0	1	0	0	2	1	1	0	1	0
142	1	—	—	1	0	1	1	0	—	3	1	1	1
135	0	0	—	1	1	0	0	2	3	4	1	1	0
143	1	—	—	1	0	1	1	0	—	3	1	1	1
144	1	—	—	1	1	1	0	0	—	3	0	0	0
145	1	—	—	1	0	0	0	0	—	2	0	0	1
146	1	—	—	1	1	1	1	0	—	3	1	1	1
147	0	1	1	1	1	1	0	0	—	3	1	0	1
148	0	1	0	0	1	1	0	0	—	3	1	1	1
149	1	—	—	1	1	1	0	2	3	2	0	0	1
150	1	—	—	1	1	1	0	0	—	1	1	0	1
151	1	—	—	1	0	0	0	0	—	2	1	1	1
152	0	1	1	1	1	1	0	0	—	3	1	1	1
153	1	—	—	1	1	0	0	1	—	4	1	0	1
154	0	0	—	1	1	1	0	0	—	0	1	1	0
155	0	1	1	1	1	1	1	0	—	4	1	1	1
156	1	—	—	1	0	1	0	0	—	4	1	0	1
157	0	1	1	1	1	1	0	0	—	3	1	1	1
158	1	—	—	1	1	0	0	0	—	3	0	1	1
159	1	—	—	1	0	1	0	0	—	4	1	0	1
160	0	0	—	1	1	1	0	0	—	4	1	0	1
161	1	—	—	1	1	1	0	0	—	4	1	0	1
162	0	0	—	1	1	1	0	0	—	4	1	0	1
163	0	0	—	1	0	1	0	0	—	4	1	0	1
164	1	—	—	1	1	0	1	0	—	3	1	0	1
165	0	1	1	1	0	1	0	0	—	3	1	1	0
166	0	1	1	1	0	1	0	0	—	1	1	1	1
167	1	—	—	0	0	0	1	0	—	3	1	1	0
168	1	—	—	1	1	1	1	0	—	4	0	0	0
169	0	1	1	1	0	1	0	0	—	3	1	1	1
170	1	—	—	0	0	0	0	0	—	3	0	1	0
171	0	0	—	1	1	1	1	0	—	4	1	1	1
172	0	0	—	1	1	1	0	0	—	3	1	1	1
172	0	0	—	1	1	1	0	0	—	3	1	1	1
173	0	1	0	1	1	1	0	0	—	4	1	1	1
174	0	1	1	1	1	1	0	0	—	4	1	0	0
175	1	—	—	1	1	1	0	0	—	4	1	0	1
176	1	—	—	0	1	1	0	0	—	3	0	0	1
177	1	—	—	1	1	1	0	2	0	3	0	0	1
178	1	—	—	1	0	0	0	0	—	4	1	0	0
179	0	1	1	0	0	0	0	0	—	3	1	0	0
180	1	—	—	0	0	0	0	0	—	4	0	0	1
181	1	—	—	1	0	1	0	0	—	4	1	0	1
182	0	0	—	1	1	0	1	0	—	4	1	1	1
183	1	—	—	1	1	1	1	0	—	0	0	1	1
184	1	—	—	1	1	1	1	0	—	4	1	0	1
185	1	—	—	1	1	1	0	2	0	1	0	1	1
186	0	0	—	1	1	1	0	0	—	3	1	1	1
187	0	1	0	1	1	1	0	0	—	3	0	1	1
188	1	—	—	1	0	1	1	0	—	3	1	1	1
189	1	—	—	1	0	0	0	0	—	3	1	1	1
190	0	1	1	1	1	0	1	0	—	1	0	1	1
191	1	—	—	1	1	1	0	0	—	4	1	0	1
192	1	—	—	0	0	1	0	0	—	1	1	0	1
193	0	0	—	1	0	1	0	0	—	4	1	1	1
194	1	—	—	1	1	1	0	0	—	3	1	1	0
195	1	—	—	0	1	1	1	0	—	3	0	1	1
196	1	—	—	1	1	1	1	0	—	4	1	0	1
197	1	—	—	1	1	1	1	0	—	3	1	0	1
198	1	—	—	0	0	1	0	1	0	3	1	1	0
199	1	—	—	0	0	0	1	0	—	4	0	1	0
200	1	—	—	0	1	0	0	0	—	2	0	1	0
201	1	—	—	1	1	1	0	0	—	2	0	1	1
202	0	1	1	1	1	1	1	0	—	4	1	0	1
203	0	0	—	1	1	1	1	0	—	4	1	1	1
204	0	0	—	1	1	1	0	0	—	4	1	1	1
205	1	—	—	1	0	1	0	1	0	1	1	0	1
206	1	—	—	1	0	0	0	0	—	4	1	0	0
207	0	1	0	0	1	1	1	0	—	1	1	1	0
208	1	—	—	1	1	0	1	0	—	4	1	0	0
209	0	1	1	1	0	0	0	0	—	3	1	1	1
210	1	—	—	1	1	1	1	0	—	3	1	1	1
211	0	0	—	1	1	1	0	0	—	0	0	1	1
212	1	—	—	1	1	1	1	0	—	3	0	1	0
213	0	1	1	1	0	1	0	0	—	4	1	1	1
214	0	1	1	1	0	1	1	2	—	3	1	1	1
215	0	—	—	1	1	1	1	1	0	4	0	0	1
216	0	1	—	0	1	1	0	1	4	0	0	1	0
217	1	—	—	0	1	1	0	0	—	1	0	1	1
218	1	—	—	1	1	1	0	0	—	3	1	0	1
219	0	1	1	1	1	1	0	0	—	4	0	0	0
220	1	—	—	0	0	0	1	1	0	1	0	0	0
221	1	—	—	1	1	1	1	0	—	3	1	0	1
222	0	1	0	0	1	1	0	2	3	2	1	1	0
223	0	1	1	0	0	0	0	0	—	2	0	0	0
224	1	—	—	1	1	1	0	0	—	3	1	0	1
225	1	—	—	1	1	1	0	0	—	4	1	0	1
226	0	1	0	1	0	0	1	0	—	1	1	1	0
227	1	—	—	1	1	0	1	0	—	4	0	1	0
228	0	1	1	1	0	1	1	1	2	3	1	0	1
229	1	—	—	1	0	0	0	0	—	4	1	0	1
230	0	0	—	1	0	1	0	0	—	3	1	1	1
231	1	—	—	1	1	1	1	0	—	2	1	1	0
232	0	1	0	1	1	1	0	0	—	3	1	1	0
233	0	0	—	1	1	1	0	0	—	2	1	0	1
234	1	—	—	1	1	1	0	0	—	4	1	0	1
235	1	—	—	1	1	0	0	0	—	4	1	0	1
236	0	1	1	1	1	0	1	0	—	3	0	1	0
237	1	—	—	1	0	1	1	0	—	4	1	0	1
238	0	0	—	1	1	1	0	0	—	3	1	0	0
239	1	—	—	1	1	0	1	0	—	3	1	1	1
240	1	—	—	1	0	1	0	0	—	4	1	1	1
241	0	1	1	1	1	0	0	2	3	4	1	0	1
242	0	0	—	1	0	1	0	0	—	3	1	1	0
243	1	—	—	1	1	1	0	0	—	3	1	1	1
244	1	—	—	1	1	1	1	0	—	3	0	1	1
245	1	—	—	1	1	1	1	0	—	3	1	1	0
246	1	—	—	1	1	1	0	0	—	4	0	1	0
247	0	0	—	1	0	1	0	0	—	3	1	1	1
248	1	—	—	0	0	1	0	1	0	4	1	0	1
249	1	—	—	1	1	0	1	0	—	3	1	1	1
250	1	—	—	1	0	1	1	0	—	3	1	1	1
251	1	—	—	1	1	1	1	0	—	3	0	0	1
252	0	1	1	1	1	1	0	0	—	3	0	1	0
253	0	1	1	1	1	1	0	0	—	4	0	0	1
254	1	—	—	1	0	0	0	1	2	1	1	1	0
255	1	—	—	1	1	1	0	0	—	4	0	1	1
256	0	0	—	1	1	1	1	0	—	3	1	0	1
257	0	1	1	1	1	1	0	0	—	3	1	0	1
258	1	—	—	1	1	1	1	0	—	4	1	0	1
259	0	1	1	1	1	1	1	0	—	4	1	1	1
260	0	1	0	0	0	0	1	0	—	3	1	1	1
261	0	1	0	1	1	1	1	1	0	3	1	1	0
262	0	1	1	1	1	0	0	0	—	3	1	1	1
263	0	1	1	1	1	1	0	0	—	4	1	0	1
264	1	—	—	1	0	1	1	0	—	4	1	0	1
265	0	1	0	1	1	1	0	0	—	3	0	1	1
266	0	1	1	1	0	1	0	0	—	4	1	0	0
267	0	1	1	1	1	1	0	0	—	1	1	1	1
268	1	—	—	0	1	1	0	0	—	3	1	1	1
269	0	0	—	0	1	0	0	0	—	3	1	0	1
270	1	—	—	1	0	1	0	0	—	4	1	0	1
271	1	—	—	1	1	1	1	0	—	4	1	0	1
272	1	—	—	1	0	1	0	0	—	3	0	1	1
273	1	—	—	1	0	1	0	0	—	4	1	0	1
274	1	—	—	0	1	0	0	0	—	2	1	1	1
275	0	1	1	1	1	1	0	0	—	3	1	0	0
276	1	—	—	1	1	1	0	0	—	3	1	0	1
277	1	—	—	1	0	1	0	0	—	4	1	0	1
278	1	—	—	1	1	0	0	0	—	3	0	1	0
279	1	—	—	0	1	1	0	0	—	4	0	0	1
280	0	1	0	1	0	0	0	0	—	3	1	0	1
281	1	—	—	1	1	0	0	1	0	3	1	0	1
282	0	0	—	1	1	1	0	0	—	4	1	1	1
283	0	0	—	1	0	1	0	0	—	4	1	0	1
284	0	1	0	0	1	1	1	0	—	3	1	1	1
285	0	0	—	0	0	0	0	1	0	2	0	1	0
286	0	1	1	1	0	1	1	0	—	3	0	1	1
287	1	—	—	1	0	1	0	1	—	3	0	1	1
288	0	1	0	0	0	1	0	1	0	3	1	0	0
289	1	—	—	1	1	1	1	0	—	2	1	1	1
290	0	1	0	1	1	0	0	0	—	4	1	1	1
291	1	—	—	1	1	1	1	2	0	3	1	0	1
292	—	—	—	—	—	—	—	—	—	—	—	—	—
293	—	—	—	—	—	—	—	—	—	—	—	—	—

**Table 11:** **Data collected from the members of Mensa Czech Republic in the neuroinformatics laboratory** ID - id of the participant, Sex - sex of the participant, Age - age of the participant, RT-H [ms] - hands average reaction time, MH - the number of missed buttons during hands reaction time measurement, EH - the number of wrongly pressed buttons during hands reaction time measurement, RT-L [ms] - legs average reaction time, StD-L [ms] - standard deviation between responses during legs reaction time measurement, BT-L [ms] - best (shortest) time achieved during reaction time measurement, WT-L [ms] - worst (longest) time achieved during legs reaction time measurement, “—” - blank data (data were not filled in).

ID	Sex	Age	RT-H[ms]	MH	EH	RT-L[ms]	StD-L[ms]	BT-L[ms]	WT-L[ms]
1	Woman	13	493	0	0	636	127	505	999
2	Woman	13	610	0	0	627	52	539	735
3	Woman	12	489	0	0	695	360	532	1327
4	Man	17	464	0	0	441	177	374	637
5	Woman	17	423	0	0	576	392	454	2000
6	Man	16	—	—	—	—	—	—	—
7	Man	19	—	—	—	—	—	—	—
8	Woman	19	—	—	—	—	—	—	—
9	Woman	13	—	—	—	—	—	—	—
10	Man	15	—	—	—	—	—	—	—
11	Man	10	—	—	—	—	—	—	—
12	Man	16	—	—	—	—	—	—	—
13	Woman	24	—	—	—	572	371	414	2000
14	Woman	29	—	—	—	—	—	—	—
15	Man	30	—	—	—	—	—	—	—
16	Man	28	—	—	—	—	—	—	—
17	Man	23	—	—	—	—	—	—	—
18	Woman	30	—	—	—	—	—	—	—
19	Woman	24	—	—	—	—	—	—	—
20	Woman	28	—	—	—	—	—	—	—
21	Man	30	—	—	—	—	—	—	—
22	Woman	24	557	1	0	734	374	596	1000
23	Woman	38	476	1	0	541	108	426	859
24	Man	33	—	—	—	576	327	515	1333
25	Man	36	495	0	0	701	215	571	1024
26	Woman	32	601	0	0	626	80	478	799
27	Woman	40	586	0	0	819	480	518	2000
28	Man	38	533	0	0	633	390	409	2000
29	Woman	38	513	1	0	619	98	477	823
30	Man	33	—	—	—	—	—	—	—
31	Woman	34	—	—	—	—	—	—	—
32	Woman	35	—	—	—	—	—	—	—
33	Man	37	—	—	—	—	—	—	—
34	Man	57	486	1	0	560	122	441	905
35	Man	43	605	0	0	637	59	554	779
36	Man	46	533	0	0	700	—	—	—
37	Woman	48	570	0	0	750	240	497	1186
38	Man	47	621	0	0	586	161	423	1153
39	Man	42	649	1	0	1057	292	980	1322
40	Man	47	512	0	0	560	112	422	917
41	Man	45	560	0	0	823	347	581	2000
42	Man	45	—	—	—	—	—	—	—
43	Man	45	—	—	—	—	—	—	—
44	Woman	42	—	—	—	—	—	—	—
45	Man	50	—	—	—	—	—	—	—
46	Man	52	485	0	0	571	208	501	1115
47	Woman	53	574	1	0	726	197	536	1339
48	Woman	57	563	0	0	772	315	500	1788
49	Man	53	513	2	0	644	127	486	919
50	Woman	51	—	—	—	—	—	—	—
51	Man	53	—	—	—	—	—	—	—
52	Man	60	—	—	—	—	—	—	—
53	Man	66	—	—	—	—	—	—	—
54	—	—	837	1	0	954	—	837	2000
55	—	—	—	—	—	685	347	510	2000
56	—	—	—	—	—	643	221	474	1450

**Table 12:** **Questionnaire data collected from the members of Mensa Czech Republic in the neuroinformatics laboratory** ID - id of the participant, Q1-Q13 - Questions provided in Section 2.3, Q1-Q7 - 1 means “yes”, 0 means “no”, Q8 - 2 means “occasionally”, 1 means “yes”, 0 means “no”, Q9 - 0 means “up to 10 cigarettes per day”, 1 means “up to 10 cigarettes per week”, 2 means “up to 20 cigarettes per day”, 3 means “up to 10 cigarettes per month”, 4 means “20 or more cigarettes per day”, Q10 - 0 means “every day”, 1 means “once per week”, 2 means “multiple times per week”, 3 means “Occasionally”, 4 means “i don't consume any alcohol”, Q11-Q13 - 1 means “yes”, 0 means “no”, “—” - blank data (data were not filled in).

ID	Q1	Q2	Q3	Q4	Q5	Q6	Q7	Q8	Q9	Q10	Q11	Q12	Q13
1	1	—	1	1	0	1	0	0	—	4	1	0	1
2	0	1	—	1	0	1	0	0	—	4	1	0	0
3	0	1	1	0	0	0	0	0	—	4	0	—	1
4	0	1	—	1	1	1	0	0	—	4	1	0	0
5	0	1	0	0	0	0	0	0	—	3	1	1	0
6	0	1	0	0	0	0	0	0	—	3	1	0	0
7	1	—	1	1	1	0	0	0	—	3	1	1	0
8	0	1	1	1	1	1	1	0	—	4	1	0	1
9	0	0	0	1	1	1	1	0	—	3	1	1	0
10	1	—	1	1	1	0	0	0	—	3	0	0	1
11	1	—	1	1	0	1	0	0	—	4	1	0	1
12	0	1	—	1	0	1	1	0	—	4	1	0	1
13	0	1	1	1	0	1	0	0	—	1	1	1	1
14	0	1	0	1	1	1	1	0	—	3	1	0	1
15	1	—	1	0	0	0	0	0	—	2	1	1	0
16	0	1	0	1	1	0	1	0	—	2	0	0	0
17	0	1	0	1	1	0	0	0	—	1	0	1	0
18	0	1	1	1	1	1	1	0	—	4	1	1	1
19	0	1	0	1	0	0	0	2	3	3	1	1	0
20	0	0	0	1	1	1	0	0	—	3	0	1	1
21	0	1	0	1	1	1	1	0	—	3	1	1	0
22	1	—	1	1	1	1	1	0	—	1	1	0	1
23	0	1	0	1	1	1	1	0	—	2	1	0	1
24	1	—	1	1	1	1	0	0	—	1	1	0	1
25	0	1	1	1	1	1	0	0	—	2	1	1	1
26	1	—	1	1	0	1	1	0	—	3	1	1	0
27	0	1	1	1	1	1	0	0	—	3	1	1	0
28	0	1	1	1	1	1	0	0	—	3	1	1	1
29	0	1	1	1	0	1	0	0	—	4	1	1	1
30	0	1	0	1	0	0	0	0	—	3	0	1	0
31	0	1	0	1	1	1	1	0	—	1	1	1	1
32	0	1	0	0	1	0	1	0	—	3	0	1	1
33	0	1	1	0	0	0	0	0	—	4	0	1	0
34	0	0	0	1	0	1	0	0	—	3	0	0	1
35	0	1	0	1	1	1	0	0	—	1	1	1	1
36	0	1	—	1	1	1	1	0	—	2	0	1	1
37	1	1	1	1	1	1	0	0	—	3	1	0	1
38	0	1	0	0	0	1	0	1	4	2	1	1	0
39	0	1	0	1	1	1	1	0	—	3	1	1	1
40	1	—	1	1	1	1	0	0	—	4	0	1	1
41	1	—	1	1	1	0	0	0	—	1	1	0	1
42	0	1	1	1	0	0	0	0	—	4	0	1	0
43	1	—	0	1	1	1	1	0	—	3	1	1	1
44	0	1	1	1	1	1	0	0	—	4	1	1	1
45	0	1	0	1	1	1	1	2	0	1	0	1	1
46	0	1	1	0	1	0	0	0	—	3	0	1	1
47	1	—	1	1	1	1	0	0	—	3	1	0	1
48	1	—	0	1	0	1	1	0	—	4	1	0	0
49	0	0	0	1	1	1	1	0	—	1	1	1	1
50	0	1	1	1	0	1	1	0	—	3	1	0	1
51	0	1	0	1	1	1	0	0	—	3	1	1	1
52	0	1	1	1	0	0	0	0	—	3	0	1	0
53	0	0	1	1	1	1	0	0	—	3	0	0	1
54	—	—	—	—	—	—	—	—	—	—	—	—	—
55	—	—	—	—	—	—	—	—	—	—	—	—	—
56	—	—	—	—	—	—	—	—	—	—	—	—	—
